# PAMP-triggered genetic reprogramming involves widespread alternative transcription initiation and an immediate transcription factor wave

**DOI:** 10.1093/plcell/koac108

**Published:** 2022-04-11

**Authors:** Axel Thieffry, Diego López-Márquez, Jette Bornholdt, Mojgan Gholami Malekroudi, Simon Bressendorff, Andrea Barghetti, Albin Sandelin, Peter Brodersen

**Affiliations:** Department of Biology, University of Copenhagen, Copenhagen N, DK-2200, Denmark; Biotech Research and Innovation Centre, University of Copenhagen, Copenhagen N, DK-2200, Denmark; Department of Biology, University of Copenhagen, Copenhagen N, DK-2200, Denmark; Department of Biology, University of Copenhagen, Copenhagen N, DK-2200, Denmark; Biotech Research and Innovation Centre, University of Copenhagen, Copenhagen N, DK-2200, Denmark; Department of Biology, University of Copenhagen, Copenhagen N, DK-2200, Denmark; Department of Biology, University of Copenhagen, Copenhagen N, DK-2200, Denmark; Department of Biology, University of Copenhagen, Copenhagen N, DK-2200, Denmark; Department of Biology, University of Copenhagen, Copenhagen N, DK-2200, Denmark; Biotech Research and Innovation Centre, University of Copenhagen, Copenhagen N, DK-2200, Denmark; Department of Biology, University of Copenhagen, Copenhagen N, DK-2200, Denmark

## Abstract

Immune responses triggered by pathogen-associated molecular patterns (PAMPs) are key to pathogen defense, but drivers and stabilizers of the growth-to-defense genetic reprogramming remain incompletely understood in plants. Here, we report a time-course study of the establishment of PAMP-triggered immunity (PTI) using cap analysis of gene expression. We show that around 15% of all transcription start sites (TSSs) rapidly induced during PTI define alternative transcription initiation events. From these, we identify clear examples of regulatory TSS change via alternative inclusion of target peptides or domains in encoded proteins, or of upstream open reading frames in mRNA leader sequences. We also find that 60% of PAMP response genes respond earlier than previously thought. In particular, a cluster of rapidly and transiently PAMP-induced genes is enriched in transcription factors (TFs) whose functions, previously associated with biological processes as diverse as abiotic stress adaptation and stem cell activity, appear to converge on growth restriction. Furthermore, examples of known potentiators of PTI, in one case under direct mitogen-activated protein kinase control, support the notion that the rapidly induced TFs could constitute direct links to PTI signaling pathways and drive gene expression changes underlying establishment of the immune state.

## Teaser

Use of alternative promoters is widespread and important upon activation of PAMP-triggered immunity (PTI), and the transcriptional PTI response is much faster than previously thought.

IN A NUTSHELL **Background:** Upon sensing an attempted infection, plants change from growth to defense by reshaping gene activity. However, there is not always a 1:1 relationship between gene and protein. Rather, genes may work like zip files that are unzipped to produce multiple proteins, depending on cellular states. One way of compressing genetic information is to use different starting points for transcription, thereby producing distinct RNA copies from a single locus.

**Question:** We asked two questions. (1) Does gene unzipping occur during immune activation via use of alternative starting points of transcription? (2) What is the timeline of gene activation during reprogramming? In particular, does reprogramming involve very rapidly activated genes that may affect later installments of the defense program?

**Findings:** Using Arabidopsis seedlings treated to activate immune responses, we found many examples of gene unzipping via use of alternative transcription start sites. In one case, a protein is sent from one place in the cell to another, in many cases RNA variants are made that result in much larger amounts of the encoded proteins, and in yet other cases, it looks like the cell can switch between production of proteins with opposite functions from the very same genes! We also discovered an ultra-rapid, transient wave of gene activity of outstanding interest, because it contains several genes known to regulate immune responses.

**Next steps:** Our findings open avenues of investigation not only on immune regulation, but also on processing of genetic information. How does gene unzipping contribute to maintenance of the immune state? Does the ultra-rapid wave of gene activity in fact drive the growth-to-defense transition? How does the cell control transcription to unzip genetic information?

## Introduction

Pathogen-associated molecular patterns (PAMPs) are conserved molecules or molecular assemblies that satisfy two criteria: (1) they are required for essential cellular or physiological functions of a pathogen and are therefore bound to evolve slowly and (2) they do not exist in the hosts of the pathogen (Janeway, 1989; Medzhitov and Janeway, 1997). Therefore, PAMPs constitute targets for distinguishing nonself from self by host immune receptors. Such PAMP-triggered immunity (PTI) mediated by specific receptors is crucial for pathogen defense in plants and animals (Nürnberger et al., 2004). In plants, a limited number of PAMP receptors have been identified that recognize conserved bacterial or fungal structures, such as flagellin (Felix et al., 1999), Elongation Factor Tu (EF-Tu; Kunze et al., 2004; Zipfel et al., 2006), and chitin (Kaku et al., 2006; Miya et al., 2007), or even pathogen-induced aberrant host molecules such as oligosaccharides released from fungal digestion of the plant cell wall (D’Ovidio et al., 2004).

The PAMP receptors FLAGELLIN-INSENSITIVE2 (FLS2) and EF-Tu RECEPTOR recognize conserved peptides in bacterial flagellin (flg22) (Gómez-Gómez and Boller, 2000; Chinchilla et al., 2006) and Elongation Factor Tu (elf18) (Kunze et al., 2004; Zipfel et al., 2006), respectively. These receptors contain an extracellular ligand-binding domain with leucine-rich repeats (LRRs) and a cytoplasmic serine/threonine kinase domain (Gómez-Gómez and Boller, 2000; Kunze et al., 2004; Chinchilla et al., 2006; Zipfel et al., 2006). Binding of the ligand initiates a signaling pathway that implicates a host of co-receptors, and involves mitogen-activated protein (MAP) kinase cascades (Asai et al., 2002; Chinchilla et al., 2007). Important MAP kinase substrates include transcription factors (TFs) of the WRKY class (Andreasson et al., 2005; Qiu et al., 2008; Mao et al., 2011), so named after their invariant Trp–Arg–Lys–Tyr tetrapeptide implicated in DNA binding (Eulgem and Somssich, 2007). WRKY TFs are themselves early PAMP response genes that regulate many defense genes characterized by the presence of WRKY-binding sites (W-boxes) in their promoters (Eulgem and Somssich, 2007). It has also recently been shown that PTI activation in *Arabidopsis thaliana* involves MAP kinase-dependent alternative splicing (Bazin et al., 2020) and translational reprogramming dependent on several features, including occurrence of a specific sequence element in 5′-leaders and sometimes skipping of upstream open reading frames (uORFs) in mRNAs encoding immune regulators (Pajerowska-Mukhtar et al., 2012; Xu et al., 2017).

Despite extensive gene expression profiling studies of plant PTI, a number of fundamental questions regarding the precise nature of transcriptional reprogramming and its control remain unresolved. These questions include, but are not limited to, three distinct areas. First, it remains ill-defined how early signaling events, such as MAP kinase activation that follows within ˂5 min of PAMP perception (Mészáros et al., 2006), are molecularly linked to transcriptional reprogramming that is typically measured, at the earliest, 30 min after PAMP perception (de Torres et al., 2003; Navarro et al., 2004; Zipfel et al., 2004, 2006; Ramonell et al., 2005; Moscatiello et al., 2006; Truman et al., 2006). Specifically, the time gap between MAP kinase activation and documented transcriptional responses suggests that changes in gene expression occurring earlier than 30 min may be part of PTI activation. Second, although it is now clear that alternative use of transcription start sites (TSSs) has important ramifications for gene function in plant biology (Ushijima et al., 2017; Kurihara et al., 2018), no information regarding the extent of alternative TSS usage following PTI activation is available. Third, it is unclear whether immunity-related enhancers—distal regulatory DNA regions that enhance transcription initiation—exist and are used to orchestrate the transcriptional activation of regulators and downstream response genes in Arabidopsis, as has been observed during activation of animal innate immunity (Arner et al., 2015).

Answers to those questions should be accessible by gene expression profiling using Cap Analysis of Gene Expression (CAGE). In addition to information on transcript abundance, CAGE yields TSS information at nucleotide resolution, because it involves capture of capped transcripts, and generation of ∼30-bp sequence reads immediately 3′ to the capped nucleotide (Takahashi et al., 2012). Thus, CAGE-based gene expression profiling clearly has the potential to answer questions on the possible existence of a very early PAMP-induced gene set, and on the possible use of PAMP-triggered alternative transcription initiation. Perhaps less intuitively clear is the fact that CAGE also has the potential to answer the third question on the possible existence of PAMP-triggered enhancers. This is because of recent findings that enhancers in animal cells can be identified as DNAse Hypersensitive Sites that produce so-called enhancer RNAs (eRNAs): bidirectional, short-lived transcripts, best observed upon inactivation of the RNA exosome complex (Andersson et al., 2014a; Andersson and Sandelin, 2020) which is at the core of cellular RNA processing and degradation by 3′–5′-exonucleolysis (Chlebowski et al., 2013). Because the study of enhancers in plants is still rudimentary, it is not yet clear whether eRNA-like-producing loci correspond to active enhancers. Nonetheless, we previously identified around 100 intergenic and intronic loci producing eRNA-like transcripts in unchallenged Arabidopsis seedlings using CAGE (Thieffry et al., 2020). In this case, detection of the short-lived eRNAs required inactivation of components of the nuclear exosome-mediated RNA decay pathway, achieved either by knockout mutation of the DEAD box helicase HUA ENHANCER2 (HEN2), a requisite, nucleoplasmic exosome cofactor (Lange et al., 2014), or by partial loss of function of the core exosome subunit RRP4 (Hématy et al., 2016; Thieffry et al., 2020). Thus, a search for PTI-related enhancers by an eRNA-focused approach would require inactivation of the nuclear exosome-mediated RNA degradation.

In addition to facilitating eRNA detection, an assessment of the relevance of nuclear exosomal RNA decay for PTI activation has merit on its own. In yeast, there is evidence that mechanisms of alternative transcription termination coupled to exosome-mediated nuclear pre-mRNA decay contribute to shape rapid reprogramming of gene expression in response to stress (Bresson et al., 2017), and a recent study in Arabidopsis provided an example of requirement of HEN2 for expression of a specific intracellular immune receptor (RPS6; Takagi et al., 2020), belonging to the nucleotide-binding-LRR class well known to be induced in PTI (Navarro et al., 2004).

In this study, we conducted a time course CAGE profiling experiment in Arabidopsis, in which wild-type (wt) and *hen2* knockout mutants were analyzed at 10 min after FLS2 activation by flg22, in addition to 30 min at which time the PTI response is known to be activated (Navarro et al., 2004). Two conclusions of broad significance were reached. First, TSS change is widespread in PTI-induced genes. This includes TSS changes of functional significance in regulatory genes and defense effectors. Second, PAMP-induced reprogramming of gene expression involves very rapid induction of a large fraction of the known PTI response genes. This also includes transient induction of a set of mRNAs encoding regulatory proteins enriched in TFs. Functions of many of these early response genes appear to converge on restriction of cellular growth and division. These findings add substantial insight into PAMP-triggered transcriptional reprogramming, and provide a detailed basis for the design of future studies to gain a molecular understanding of how early PTI signaling events control transcriptional reprogramming leading to establishment and maintenance of the immune state.

## Results and discussion

### Validation of PTI induction and TSS identification by CAGE

**Figure 1:**
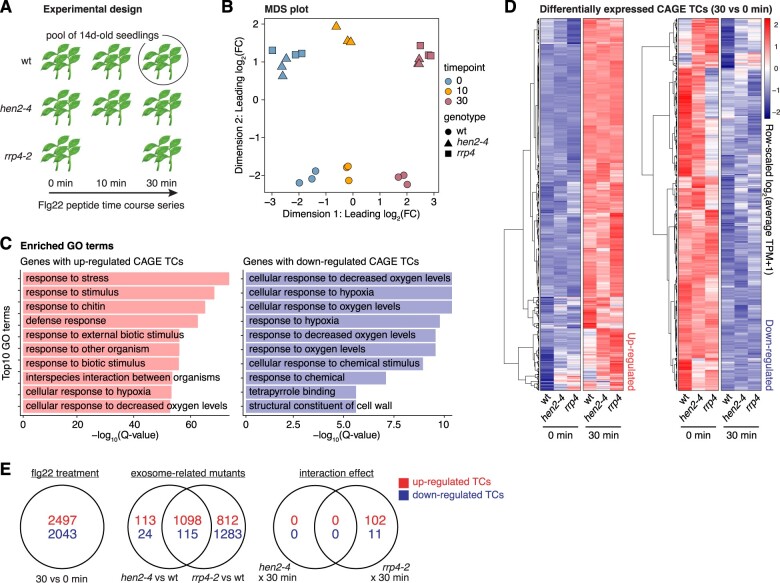
Experimental setup and validation of PTI response. A, Overview of experimental design. Pools of 14-day-old seedlings from wt and two exosome-related mutants (*hen2-4* and *rrp4-2*, see “Materials and methods”) were subjected to flg22 treatment in biological triplicates followed by CAGE library preparation and sequencing at the set time points. The *fls2* mutant was included in the time course for initial validation of the treatment. B, MDS plot. *X*- and *Y*-axes show the first two dimensions. Each point corresponds to a CAGE library, colored by time of flg22 treatment. Shapes indicate genotype. Axes are scaled as leading log_2_ FC; the root-mean-squared average of the log_2_ FC of the top 1,000 genes best separating each sample. C, Enriched GO terms of genes with CAGE TCs responding to the flg22 treatment of wt after 30 min. *X*-axis shows *P*-values after correction for multiple testing and log transformation. *Y*-axis shows the top 10 enriched terms. Terms for genes with upregulated (red) and downregulated (blue) CAGE TCs are shown in left and right panels, respectively. D, Hierarchical clustering of CAGE TCs that were significantly upregulated (left heatmap) or downregulated (right heatmap) at 30 min after flg22 treatment versus 0 min. Each row represents a CAGE TC. Color indicates row-scaled, TPM-normalized expression. E, Venn diagram of differentially expressed CAGE TCs across experimental conditions. Top (red) and bottom (blue) numbers show up- and downregulation, respectively. “*Flg22 treatment*” shows TCs responding to the flg22 induction at 30 min compared to control (0 min). “*Exosome-related mutants*” denote the comparisons of *hen2-4* and *rrp4-2* to wt samples. The “*interaction effect*” captures TCs whose response differs due to the interaction of exosome-related mutants and flg22 treatment at 30 min.

To study the PAMP-triggered immune response, we applied the flg22 peptide to seedlings in liquid medium. In addition to wt and *hen2-4* (Lange et al., 2011), we included seedlings of two different genotypes: the flg22-insensitive *fls2* mutant for validation of our experimental set-up, and a hypomorphic mutant allele of the exosome core factor RRP4 (*rrp4-2*) for CAGE profiling at 30 min to answer the additional question of whether nuclear RNA quality control mediated by the exosome machinery may play a role in PAMP-triggered reprogramming of gene expression (Figure 1A). Our flg22 treatments were effective, because known response genes such as *FLG22-INDUCED RECEPTOR-LIKE KINASE1 (FRK1)*, *MAP KINASE3* (*MPK3*), *WRKY22*, and *WRKY29* were induced in wt, while no induction could be detected in the *fls2* mutants (Supplemental Figure S1A).

We, therefore, constructed and sequenced triplicate CAGE libraries from flg22 inductions conducted in this way. Analyses of the resulting data from unchallenged samples have been reported previously (Thieffry et al., 2020). The present full data set, including flg22 induction, was treated in the same way, that is, the 5′-ends of CAGE tags located within 20 bp from each other on the same strand were clustered into CAGE tag clusters (TCs), quantified using their total number of tags, and finally normalized into tags per million (TPM; Supplemental Data Set 1).

Initial analyses showed that our CAGE data faithfully captured known TSSs (Thieffry et al., 2020), and delivered two arguments that our flg22 treatments induced global gene expression changes typical of PTI (Figure 1, B–D). First, multidimensional scaling (MDS) of CAGE TCs showed that the samples mainly clustered according to time after flg22 induction and according to their genetic background (Figure 1B). Second, differential expression analysis identified more than 2,000 upregulated CAGE TCs (log_2_ fold-change [FC] ≥ 1, FDR ≤ 0.05, see “Materials and methods”) when comparing samples harvested 30 min post treatment to untreated controls (Figure 1E; Supplemental Data Set 2). The set of genes with upregulated CAGE TCs at 30 min was highly enriched in biological processes related to stress and defense, with hallmarks of PTI (Figure 1C).

### Nuclear exosome-mediated RNA decay does not contribute substantially to PTI-associated reprogramming of gene expression

We next compared the PTI response in wt with that in *hen2-4* and *rrp4-2*. We found that the overall PTI response was very similar in wt and *hen2-4* (Figure 1D), including induction of *MPK3*, *WRKY22*, and *WRKY29* as in wt (Supplemental Figure S1B). Nonetheless, a small set of around 100 CAGE TCs was differentially expressed between wt and *rrp4-2* at 30 min posttreatment (Figure 1E; Supplemental Data Set 2). Since HEN2 is strictly nucleoplasmic, and RRP4 is a core exosome component required for both nuclear and cytoplasmic exosome functions, the most straight-forward interpretation of these results is that exosome-mediated cytoplasmic mRNA decay plays a minor role in induction of the immune state in Arabidopsis. In contrast, there is little indication for such a role of nuclear RNA quality control. These are relevant conclusions, because nuclear RNA quality control mediated by the exosome has previously been observed to have such regulatory roles in stress-induced genetic reprogramming in yeast (Bousquet-Antonelli et al., 2000; Bresson et al., 2017), as well as transcriptional control in mouse stem cells (Lloret-Llinares et al., 2018; Garland et al., 2019). We also note that the possible involvement of cytoplasmic exosomal mRNA decay is consistent with recent identification of mammalian mRNAs whose degradation depends exclusively on the cytoplasmic exosome pathway (Tuck et al., 2020). It is also consistent with our previous identification of an extensive set of highly expressed mRNAs that over-accumulates in untreated seedlings in *rrp4-2*, but not in *hen2-4* mutants, relative to wild type (Thieffry et al., 2020).

### Features of alternative TSS usage during PAMP-triggered genetic reprogramming

Alternative promoter usage may increase the repertoire of gene regulation in a number of ways. First, multiple promoters resulting in the same protein product may facilitate distinct responses to different stimuli, or jointly increase the dynamic range of expression. Second, alternative promoters may be located so that the resulting transcripts contain different RNA regulatory elements or encode distinct sets of functional protein domains or localization signals. Recent studies show that this type of regulation has profound importance in the plant response to light or sensing of light quality (Ushijima et al., 2017; Kurihara et al., 2018). Nonetheless, the extent and importance of alternative promoter usage in the PTI response are not known.

**Figure 2:**
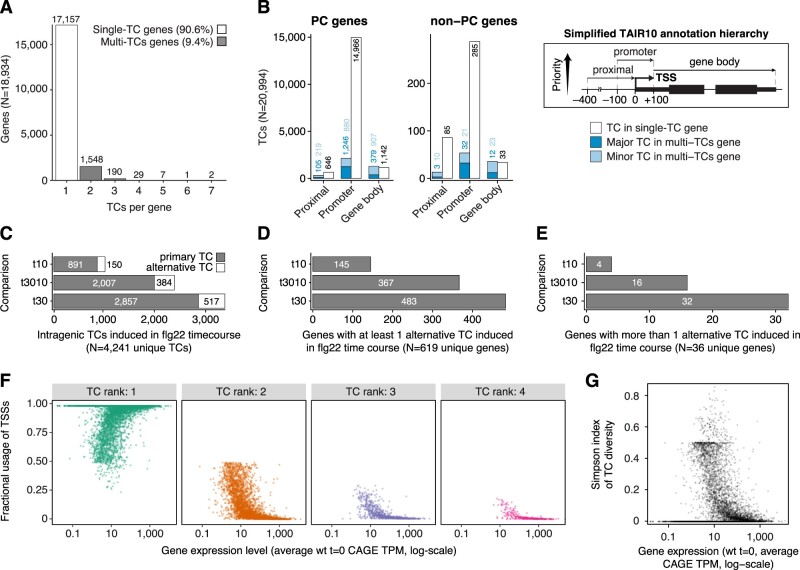
Alternative TSS usage in PTI. A, Extent of alternative TSSs. *Y*-axis shows the number of genes having a set number of TCs (*X*-axis). Only intragenic TCs contributing at least 10% to the expression of their cognate gene were considered (see “Materials and methods”). Bar colors distinguish single-TC from multi-TCs genes. B, Annotation of intragenic TCs. Number of TCs (*Y*-axis) overlapping TAIR10 genomic features (*X*-axis) based on a simplified hierarchical annotation system (right). Left parts show data for PC genes, middle parts for non-PC genes. Bar colors indicate whether the TC category originates from a single- or multiple-TCs gene, and whether TCs are the major contributor (dark shade) or minor contributors (light shade) to the expression of their cognate gene. C, *X*-axis shows the number of differentially expressed intragenic TCs in the flg22 time course. Y-axis shows the time point comparison used for differential expression analysis: t10 (10 versus 0 min), t3010 (30 versus 10 min), and t30 (30 versus 0 min). Bar colors indicate whether CAGE TCs are located within ±100 bp from the most upstream 5′-end of TAIR10 gene models (primary TC) or not (alternative TC). D and E, *X*-axis show the number of genes with at least one (D) or more than one (E) alternative TC induced during the flg22 time course. *Y*-axes are organized as in (C). F, Fractional usage of CAGE TCs (*Y*-axis) as a function of gene expression (*X*-axis, log-scaled) for multi-TCs genes. CAGE TCs are grouped according to their rank of gene expression contribution, with rank 1 representing dominant TCs, that is, the TCs contributing most to the expression of their cognate genes. Only ranks 1–4 are shown. G, Plot of Simpson index of CAGE TC diversity (*Y*-axis) against gene expression level (*X*-axis, log-scaled).

We, therefore, first considered the pan-experiment landscape of alternative TSSs by investigating the localization of intragenic CAGE TCs regardless of expression dynamics. Similar to our previous analysis on untreated seedlings (Thieffry et al., 2020), we found that most genes had only one CAGE TC: when we only considered CAGE TCs that contributed at least 10% to the expression of their cognate gene, 91% of all detected genes had only one CAGE TC (Figure 2A; Supplemental Data Set 1). This threshold of at least 10% contribution for a particular CAGE TC to be considered was used for all intragenic TCs analyzed below (see “Materials and methods”). For the majority of single-TC genes, the TCs were located within the promoter region (CAGE TC peak located ±100 bp from TAIR10 annotated TSSs) of protein-coding (PC) and noncoding genes, as expected (Figure 2B). For example, ∼90% of TCs in single-TC genes overlapped the promoter region and only ∼6% were located within gene bodies. For genes with multiple TCs, we labeled the most highly expressed TC as “major” (others as “minor”), and overlapped those with the simplified annotation as above in Figure 2B. Both major and minor TCs were most commonly observed in annotated promoter regions, but a substantial fraction was also observed within gene bodies. For example, in *PC* genes, major and minor TCs overlapped annotated promoter regions in 72% and 43% of cases, and gene bodies in 22% and 45% of cases, respectively (Figure 2B). These observations indicate that although the great majority of genes only use one TC, a considerable number of alternative TCs in multi-TC genes are found within gene bodies, motivating a more in-depth analysis of alternative TSS usage in PTI.

### Alternative TSS usage is common during PTI activation, but a substantial fraction bears hallmarks of transcriptional noise

We found that >15% of intragenic TCs differentially expressed across treatment time points did not overlap the primary annotated TSS, defined as the most upstream promoter from TAIR10 (±100 bp). These TCs could therefore be considered as manifestations of changed alternative promoter activity during the time course (Figure 2C). A total of 619 genes had at least one such alternative TC that was differentially expressed between at least one pair of time points, where the 30 to 0 min comparison had the highest number of differentially expressed alternative TCs (Figure 2D). A small set of 32 genes even had two or more alternative TCs differentially expressed in the comparison of samples treated for 30 min versus untreated samples (Figure 2E). Thus, the use of alternative TSSs is a common feature of the transcriptional PTI response, prompting a more detailed analysis of its consequences by identification of cases with particular potential for functional relevance.

A recent re-analysis of human and mouse transcriptome data concluded that the majority of alternative transcription initiation events is likely to represent transcriptional noise, mainly because it is predominantly observed when genes are lowly expressed: under conditions of higher gene expression, genes tend to use fewer TSSs, and the relative use of the dominant TSS increases (Xu et al., 2019). We analyzed the set of alternative TCs identified in untreated seedlings for these trends, and found that a majority of them indeed follows this same pattern: the fractional usage of the dominant TSS (rank 1) increases with expression level (Figure 2F), while the fractional usage of minor TSSs (ranks 2–4 shown in Figure 2F) decreases with expression level. Consistent with these patterns, the TC diversity decreases with gene expression level, as measured by the Simpson’s diversity index (Figure 2G). Thus, rigorous criteria must be applied to identify and validate cases of alternative transcription initiation of functional relevance.

We, therefore, chose a conservative expression criterion in which a TC must account for at least 10% of the CAGE tags mapping to a gene to be considered. For those cases chosen for further analysis, we included additional steps to (1) validate that the CAGE-seq data identifies genuine alternative transcription initiation sites (see below), (2) test whether RNA species defined by alternative TSSs are substrates of the nuclear exosome, a common feature of nonfunctional transcripts (Andersson et al., 2014b), through the use of our CAGE-seq data obtained in the *hen2* mutant background, (3) probe the existence of polyadenylated mRNA species matching the mapped TSS sites in existing full-length cDNA collections and transcriptome annotations (Seki et al., 2002; Ivanov et al., 2021), and (4) examine evidence for translation of identified mRNA isoforms by mining published ribosome-profiling (Ribo-seq) data (Hsu et al., 2016; Bazin et al., 2017; Xu et al., 2017; Yoo et al., 2020; Supplemental Data Set 3).

For validation of TSS sites, we used 5′-rapid amplification of cDNA ends (5′-RACE). CAGE and 5′-RACE use different biochemical principles for inference of cap presence: while CAGE relies on reaction of biotinylated hydrazine with aldehydes generated by HIO_4_-mediated oxidation of 2′–3′ vicinal diols in the cap nucleotide, 5′-RACE uses RNA adaptor ligation to 5′-phosphates generated by pyrophosphatase treatment after elimination of preexisting free 5′-phosphates by alkaline phosphatase treatment. Thus, confirmation by 5′-RACE adds information to the CAGE experiment, because it excludes the possibility that an internal abasic site, for example, generated by oxidative damage, gives rise to CAGE signal as a consequence of hydrazine reaction with the 1′-carbon atom in dynamic equilibrium between aldehyde and hemi-acetal forms.

### Promoter switching during PTI

**Figure 3:**
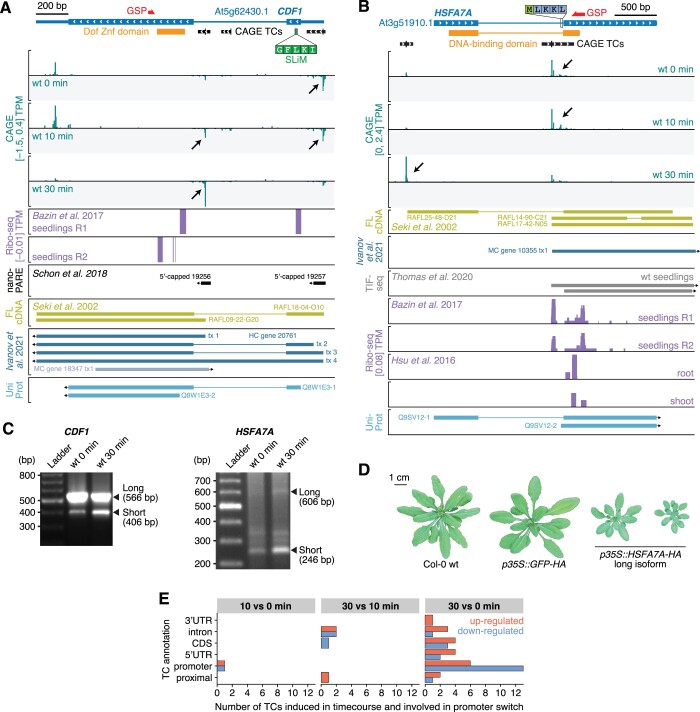
Examples of promoter switches in PTI. A, Genome browser view of the *CDF1* gene. The TAIR10 gene model is shown on top with large and thin blue blocks indicating coding regions and untranslated regions, respectively. Blue lines represent introns. White arrows indicate direction of transcription. Protein domains are shown in orange. CAGE TC are shown as black blocks with the tick marking the TC peak position. The SLiM in *CDF1* required for TOPLESS binding is indicated by a green block, along with its amino acid sequence. The 5′-RACE GSP used in (C) is marked by a red arrow. Bottom tracks show CAGE signal expressed in average TPMs across wt replicates for 0, 10, and 30 min following flg22 treatment in green. Negative CAGE signal indicates antisense initiation of transcription. Violet tracks show Ribo-seq signal in two seedling replicates (Bazin et al., 2017). Black arrows highlight important changes in CAGE TCs usage during the time course (see main text). The black track shows 5′-capped transcripts detected by nano-PARE by Schon et al. (2018). Evidence from full-length cDNAs (FL-cDNA; Seki et al., 2002) is shown in the gold track, followed by transcriptome annotation in dark (sense) and light blue (antisense) from Ivanov et al. (2021), and UniProt proteins in sky blue. B, Genome browser view of the *HSFA7A* gene, organized as in (A), with one supplementary track showing transcript isoform sequencing (TIF-seq) data from Thomas et al. (2020). C, PCR fragments obtained by 5′-RACE with CDF1 and HSFA7A GSPs (see (A) and (B)). Reference sizes are indicated on the left side in bp (Ladder). First and second samples are used as input RNA from wt seedlings at 0 and 30 min after flg22 treatment, respectively (see “Materials and methods”). Fragments corresponding to the long and short transcript isoforms (based on alternative transcription initiation events detected by CAGE) are indicated by black arrows on the right side. D, Rosette phenotypes of independent transgenic lines constitutively expressing the HSFA7A long isoform or GFP, and of nontransgenic Col-0 wt Arabidopsis plants, as indicated. E, Annotation of CAGE TCs involved in promoter switches during flg22 time course. *X*-axis shows the number of TCs falling in each annotation category (*Y*-axis, see Figure 2B, right). Bar colors indicate downregulation (blue) or upregulation (red) for each of the time course comparisons (columns).

We initiated our search for functionally important alternative transcription initiation during PTI activation by looking for TSS switching in which pairs of TCs are significantly differentially expressed (log_2_ FC ≥ 1, FDR ≤ 0.05, see “Materials and methods”) in opposite directions over time. A small set of 21 genes satisfied this criterion (Supplemental Data Set 4). The genes *CDF1* (At5g62430) encoding the TF CYCLING DOF FACTOR1, and *HSFA7A* (At3g51910) encoding the HEAT SHOCK TRANSCRIPTION FACTOR A7A provide compelling examples of TSS switches likely to have functional impact. Both genes produce short and long isoforms whose presence as functional, PC mRNAs is well supported by a variety of transcriptomic data (Figure 3, A and B), including insensitivity to inactivation of *HEN2* (Supplemental Figure S2), existence of full-length cDNAs matching the CAGE-defined TSSs, and Ribo-seq peaks close to predicted start codons (Figure 3, A and B). Furthermore, 5′-RACE analysis recapitulated the dynamics of TSS use during PTI activation in both cases (Figure 3C).

In *CDF1*, the switch from an upstream to a downstream TSS during PTI induction favors production of an mRNA encoding a protein lacking an N-terminal short linear motif (SLiM, IKLFG) required for interaction with the transcriptional co-repressor TOPLESS (Goralogia et al., 2017) (Figure 3A). The TSS switch in *CDF1* is, therefore, predicted to change CDF1 function profoundly during PTI activation, potentially switching from repressor to activator. At the *HSFA7A* locus, transcription switches from a TSS in the first intron, downstream of the exon that encodes the DNA-binding domain of HSFA7A, to a TSS slightly upstream of the annotated promoter region (Figure 3B). Thus, in this case, PTI-induced TSS switching results in repression of a protein product with oligomerization, but not DNA-binding properties, potentially capable of interfering with HSF function, because of their requirement for oligomerization for DNA binding (Guo et al., 2016), and in de novo production of a functional, DNA-binding TF. Indeed, constitutive expression of the long *HSFA7A* isoform was sufficient to restrict growth substantially (Figure 3D; Supplemental Figure S3), suggesting that promoter switching at the *HSFA7A* locus contributes to maintenance of the growth-restricted immune state. This is consistent with the described importance of members of the HSF family in plant stress adaptation (Guo et al., 2016), including elf18-induced PTI (Pajerowska-Mukhtar et al., 2012). Overall, TSS switching events, often from a downregulated TC in the promoter region to an upregulated TC localized downstream, were mostly observed after 30 min of flg22 treatment (Figure 3E), suggesting that TSS switching generally may play a role in maintenance of the immune state rather than its establishment.

### Alternative TSS usage affecting protein domain composition

**Figure 4:**
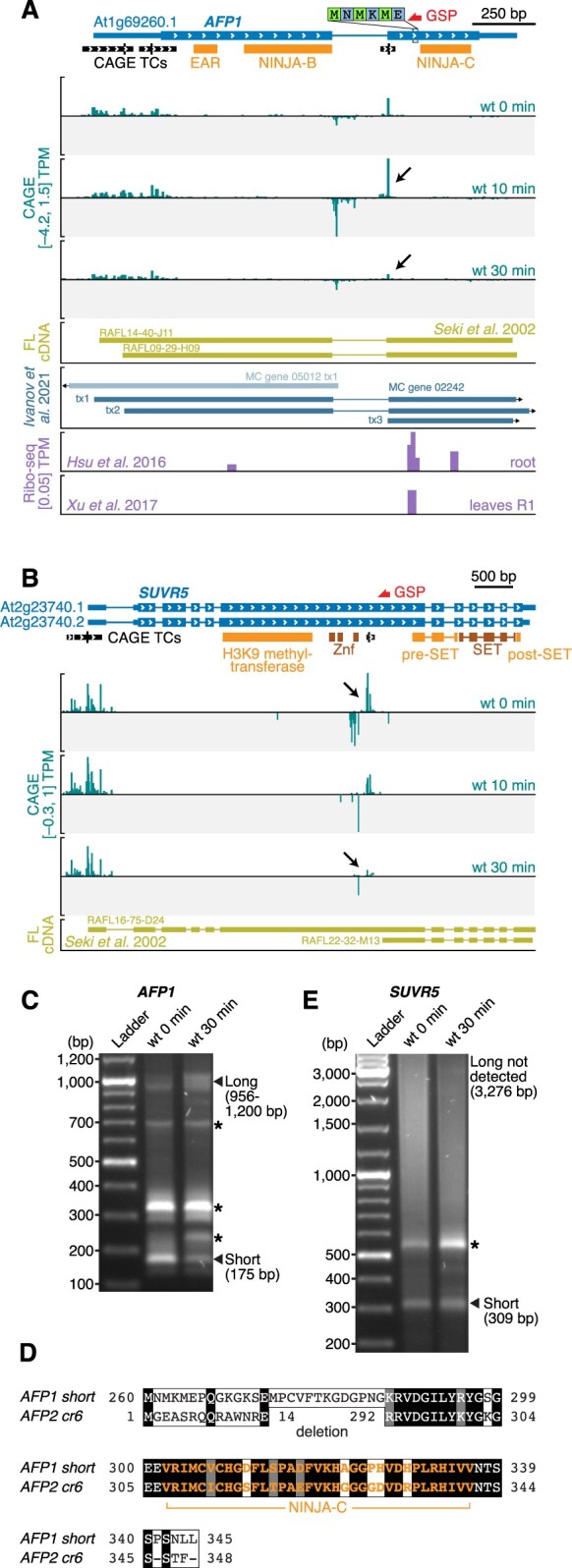
Examples of alternative TSS usage affecting protein domain composition. A and B, Genome browser views of *AFP1* (A) and *SUVR5* (B), organized as in Figure 3A. C and E, Separation of PCR fragments obtained by 5′-RACE with primers specific for AFP1 (C) and PUS5 (E), organized as in Figure 3C. D, Sequence alignment of the microproteins resulting from translation of the naturally occurring short *AFP1* mRNA isoform and of the *AFP2* mRNA generated from the in-frame *afp2*-*cr6* deletion demonstrated experimentally to be biologically functional (Hong et al., 2020). The NINJA-C domain is indicated in orange.

Inspired by the examples of promoter switching above, we conducted an extended analysis of TCs whose localization would predict disruption or exclusion of protein content. We found that 454 intragenic CAGE TCs in our dataset were localized within or downstream of protein domains (in total, this corresponded to 428 genes). Of these, 127 TCs within 125 genes were differentially expressed during the time course, including *AFP1* (*ABI5 BINDING PROTEIN*, At1g69260) encoding a transcriptional co-repressor and *SUVR5* (At2g23740) encoding a histone H3 lysine 9 (H3K9) methyl transferase (Figure 4, A and B; Supplemental Data Set 5). AFP1 and SUVR5 have both been implicated as repressors of gene expression with importance in environmental adaptation. Mutation of *AFP1* causes abscisic acid hypersensitivity and reduced salt stress resistance (Garcia et al., 2008), while mutants in *SUVR5* display de-repressed expression of genes with gene ontology (GO) term “Response to stimulus,” including stress- and auxin-response genes (Caro et al., 2012).

Both *AFP1* and *SUVR5* showed similar expression dynamics during our PTI activation: a CAGE TC corresponding roughly to the annotated TSS giving rise to an mRNA encoding a full-length protein is constitutively expressed, while a downstream TC is strongly repressed upon PTI activation (Figure 4, A and B). All TCs are insensitive to *HEN2* mutation (Supplemental Figure S2), pointing to a function of the detected transcripts. Indeed, the existence of short and long isoforms as functional mRNAs is supported by full-length cDNAs matching the TSSs defined by CAGE in the case of *SUVR5* (Figure 4B), and by medium-confidence transcripts annotated based on many sources of Arabidopsis transcriptomic data in the case of *AFP1* (Figure 4A; Ivanov et al., 2021). In addition, 5′-RACE successfully detected the short isoforms of both genes (Figure 4, C and E).

For both *AFP1* and *SUVR5*, the downstream TSS directs production of an mRNA encoding a single protein domain, a so-called microprotein, which may interfere with the function of the full-length multi-domain protein. Such a functional switch via repression of a dominant interfering microprotein during PTI activation is particularly likely in the case of AFP1. The repression of the short AFP1 mRNA by flg22 treatment was confirmed by 5′-RACE (Figure 4C), and the unique appearance of Ribo-seq signal at the start codon of the short form in untreated tissues suggests that it can be translated into a microprotein comprising only the C-terminal domain, but neither the Ethylene-responsive binding factor-associated Amphiphilic Repression (EAR) nor Novel Interactor of JAZ (NINJA) domains with transcriptional co-repressor function (Figure 4A; Ohta et al., 2001; Pauwels et al., 2010). This AFP1-derived microprotein is nearly identical to the C-terminal domain of AFP2 (At1g13740; Figure 4D) that was recently shown to be functionally equivalent to the naturally occurring microprotein LITTLE NINJA (LNJ) in monocots (Hong et al., 2020). LNJ expression causes constitutive jasmonic acid (JA) responses, probably by interfering with homodimerization of the adaptor protein NINJA that is essential for linking core JA response regulators to the TOPLESS transcriptional co-repressor (Pauwels et al., 2010; Hong et al., 2020). Our analyses indicate that alternative transcription initiation at the Arabidopsis *AFP1* locus leads to production of a naturally occurring LNJ-type microprotein encoded by an mRNA that is rapidly repressed upon PTI induction. Given the proven potential of LNJ to facilitate JA signaling (Hong et al., 2020), and the promotion of bacterial pathogenesis by virulence factor-mediated ectopic JA responses (Zheng et al., 2012; Gimenez-Ibanez et al., 2014; Nakano and Mukaihara, 2019), it is tempting to speculate that LNJ repression at the *AFP1* locus enhances the repression of JA responses as part of PTI establishment.

It is a curious property of the microprotein-encoding *AFP1* and *SUVR5* mRNAs that their transcription is accompanied by a divergent noncoding transcript detectable even in wt with exosome activity (Figure 4, A and B). Our previous analysis on the transcriptional output in Arabidopsis found that such cases are rare (Thieffry et al., 2020), perhaps indicating that special mechanisms of transcription initiation operate at the intragenic TSSs in *AFP1* and *SUVR5*.

For SUVR5, the putative microprotein contains only pre- and post-SET domains involved in substrate binding and regulation of catalysis, but neither of the Zinc finger domains required for DNA binding, nor the actual H3K9 methyltransferase domain (Caro et al., 2012; Figure 4B). Taken together, our analysis of alternative transcription initiation affecting protein domain composition highlights several cases of rapid PTI-induced repression of microproteins with proven (AFP1/LNJ) or predicted (HSFA7A, SUVR5) potential to interfere with the function of multidomain proteins that share at least one domain with the dynamically expressed microproteins.

### Alternative TSS usage affecting N-terminal target peptides

To catalog transcription initiation events affecting the occurrence of predicted target peptides for entry into the secretory pathway, or for plastidial or mitochondrial import, we first scanned the complete set of TAIR10 proteins with SignalP-5.0 and TargetP-2.0 (Almagro Armenteros et al., 2019) (see “Materials and methods”) and identified 6,985 proteins with a predicted localization signal (Supplemental Data Set 6). Among those, 4% (284/6,985) had at least one intragenic CAGE TC (subjected to the same thresholds as above) located within or downstream of the sequence encoding a predicted target peptide. Notably, 78 of those TCs (within 76 genes) were differentially expressed in the flg22 time course (Supplemental Data Set 6).

To further analyze the occurrence of differential exclusion of N-terminal target peptides, we identified intragenic TCs whose response in the time course differs from that of other TCs within the same gene (see “Materials and methods”). Clear cases of such differential TC usage (DTU) involving partial or total exclusion of a localization signal were identified in 26 genes, where one promoter produced a transcript isoform with and another without the target peptides (Supplemental Data Set 6).

**Figure 5:**
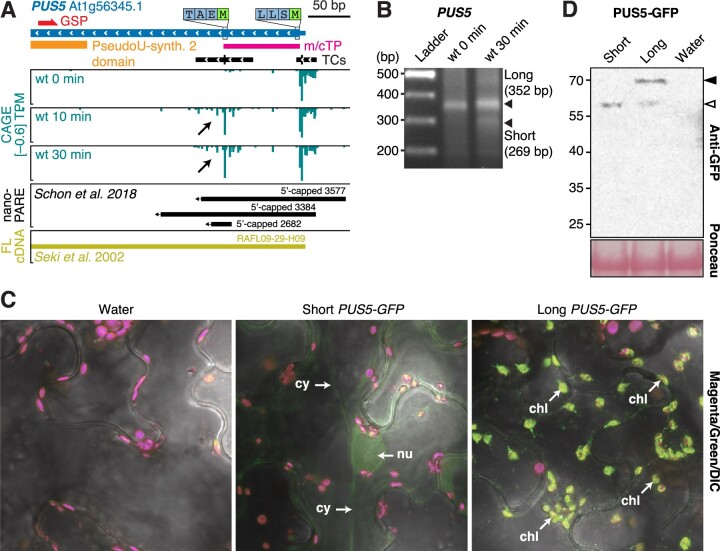
Alternative TSS usage at the *PUS5* locus determines the subcellular localization of the PUS5 protein. A, Genome browser view of *PUS5*, organized as in Figure 3A. The predicted mitochondrial/chloroplast target peptide (m/cTP) is indicated by a pink block. B, Separation of PCR fragments obtained by 5′-RACE with primers specific for PUS5, organized as in Figure 3C. The migration of the entire flg22 30 min lane in the gel with PUS5 RACE PCR products is shifted upward, explaining the apparent misalignment of the short product (269 bp) with the ladder. C, Confocal microscopy images of transiently expressed short and long *PUS5* isoforms fused to *GFP* in *N. benthamiana* leaves. Signals in magenta and green channels are overlaid with the differential interference contrast microscopy images. Nu, nucleus; cy, cytoplasm; chl, chloroplast. Images from the individual channels are shown in Supplemental Figure S4 to support the identification of structures harboring GFP signal in long *PUS5-GFP* as chloroplasts, and to support the conclusion that signal in the green channel from chloroplasts obtained with cells expressing short *PUS5-GFP* does not exceed that obtained with cells that do not express *GFP*. D, Immunoblot of *PUS5*-*GFP* isoforms transiently expressed in *N. benthamiana* leaves. Protein extracts were prepared at 2 days postinfiltration and analyzed by immunoblotting using anti-GFP antibody. White arrow indicates the short/processed isoform. Black arrow indicates the long isoform. Ponceau staining of the membrane is shown as loading control.

To show the importance of this type of alternative transcription initiation, we focused on the different isoforms expressed from the *PUS5* gene (At1g56345; Figure 5A) that encodes a stand-alone pseudouridine synthase, that is, an enzyme that catalyzes uridine-pseudouridine isomerization in RNA in a small nucleolar RNA-independent manner. PUS5 constitutively expresses a long isoform, and a short isoform is robustly and rapidly induced by flg22 (Figure 5A). The abundance of both isoforms were unaffected by mutation of *HEN2* (Supplemental Figure S2), and their existence was confirmed by detection of 5′-capped species matching those observed by CAGE by both nanoPARE (Schon et al., 2018) and 5′-RACE, the latter also confirming the induction of the short form by flg22 (Figure 5, A and B).

In contrast to the constitutively expressed, annotated TSS, the mRNA resulting from transcription initiation at the induced downstream TC does not include the sequence encoding a mitochondrial/plastidial transit peptide (Figure 5A), but does retain coding potential for the pseudouridine synthase catalytic domain. We used transient expression of *PUS5-GFP* fusions in *Nicotiana benthamiana* to show that the long and short PUS5 mRNA isoforms indeed encode proteins with distinct subcellular localization: the long form was clearly detected in chloroplasts and, probably, mitochondria, while the short form accumulated at lower levels in the nucleus and in the cytoplasm (Figure 5, C and D; Supplemental Figure S4). Stand-alone pseudouridine synthase-catalyzed introduction of pseudouridines is well established in tRNAs and spliceosomal snRNAs (Spenkuch et al., 2014), but has also recently been found in mRNAs (Borchardt et al., 2020) in several eukaryotic organisms, including plants (Sun et al., 2019). Since pseudouridylation of tRNA can be important for translation of specific mRNAs encoding regulatory factors (Cui et al., 2021) and snRNA pseudouridylation is linked to growth habit and stress adaptation in yeast, presumably via effects on alternative splicing (Morais et al., 2021), the use of alternative PUS5 isoforms with distinct subcellular localization could have important ramifications for PTI-associated genetic reprogramming.

### TSS change resulting in transcripts differing in uORF content

**Figure 6:**
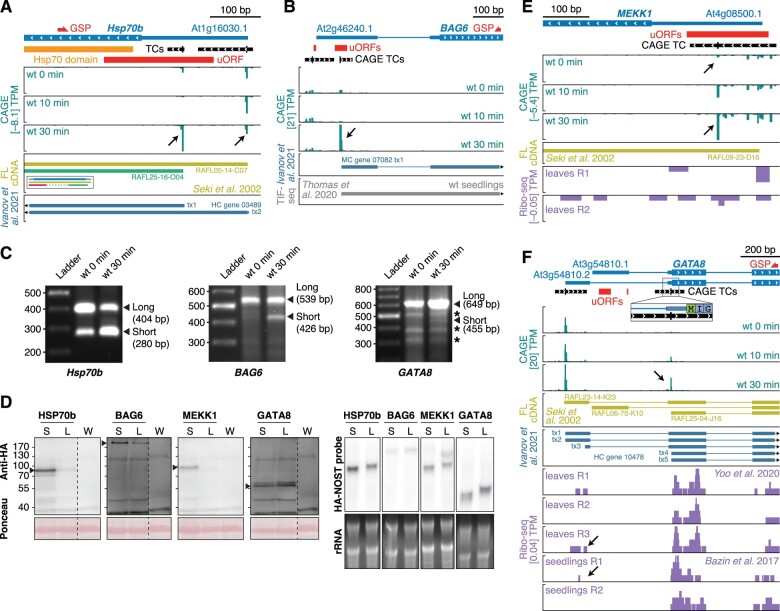
Examples of alternative TSSs influencing the presence of uORFs. A, B, E, and F, Genome browser views for *HSP70b* (A), *BAG6* (B), *MEKK1* (E), and *GATA8* (F) genes, organized as in Figure 3A, with red blocks indicating uORFs. C, Separation of PCR fragments obtained by 5′-RACE with GSPs for *Hsp70b* (left), BAG6 (middle), and *GATA8* (right), organized as in Figure 3C. D, Left: Immunoblot analysis of C-terminally HA-tagged fusions of HSP70b, BAG6, MEKK1, and GATA8 long (L) and short (S) isoforms transiently expressed in *N. benthamiana* leaves. Protein extracts were prepared at two days postinfiltration and analyzed by immunoblotting using anti-HA antibody. Ponceau staining of the membrane is shown as loading control. W indicates water-infiltrated negative control. Black arrows indicate bands of interest. All samples were loaded on the same gel with one negative control lane. Signals for individual genes have been cropped for presentation purposes, and the single negative control lane applicable to all samples is duplicated in each of the subpanels in (D). Long and short exposures of the uncropped blots are shown in Supplemental Figure S5. Right: RNA gel blot analysis of total RNA extracted from samples analyzed in (D). Ethidium bromide staining is used as loading control. A probe against the HA tag and NOS terminator was used for hybridization.

Translational control is emerging as a major theme in regulation of plant stress responses (Xu et al., 2017; Kurihara et al., 2018). Transcript quality is important for the efficiency of mRNA translation, and recent studies have indicated that uORFs are implicated in control of gene expression during the activation of PTI as well as other stress responses (Xu et al., 2017). Since alternative TSS usage can lead to inclusion or exclusion of uORFs in mRNAs (Kurihara et al., 2018), we specifically searched for cases that would lead to production of such alternative mRNAs upon flg22 induction. Using the same approach as above, we found 510 TCs in 480 genes that were within or downstream of uORF regions and differentially expressed during the flg22 time course (Supplemental Data Set 7). For example, chaperone induction is associated with PTI (Navarro et al., 2004), and our analyses show that during PTI activation, the genes encoding both an Hsp70 isoform (At1g16030 and HSP70b) and BAG6 (Bcl2-associated athanogene 6, At2g46240), an Hsp70 nucleotide exchange factor, use downstream TSSs excluding one and two uORFs in their mRNAs, respectively (Figure 6, A and B). In both cases, the existence of long, uORF-containing and shorter uORF-free mRNA forms as well as their expression dynamics during PTI activation were confirmed by 5′-RACE (Figure 6C), and for *HSP70b*, full-length cDNAs corresponding to both long and short forms were identified (Figure 6A). Furthermore, the abundance of all *HSP70b* and *BAG6* isoforms was unaffected by *HEN2* mutation (Supplemental Figure S2).

We used transient expression in *N.* *benthamiana* to test the properties of long and short isoforms with regards to protein production. Remarkably, at comparable mRNA levels, the short *HSP70b* and *BAG6* isoforms caused production of substantially higher levels of protein (Figure 6D) than their long counterparts, indicating that chaperone induction during PTI involves not only increased mRNA levels, but also, probably more importantly, changed mRNA quality through alternative transcription initiation.

We also considered the gene encoding the important signal transducer MEKK1 (At4g08500), a MAP kinase kinase kinase required for activation of the MPK4 cascade by flg22 (Ichimura et al., 2006; Suarez-Rodriguez et al., 2007). Here, the TSS distribution within its sole CAGE TC shifts during PTI activation so that an mRNA free of uORFs becomes predominant (Figure 6E). Previously published Ribo-seq data support association of the uORFs in MEKK1 mRNAs with long leaders with ribosomes (Figure 6E; Xu et al., 2017), arguing for a genuine function in repressing translation of the main MEKK1 ORF. Indeed, in the transient expression assay in *N.* *benthamiana*, the short MEKK1 mRNA isoform induced during PTI produced substantially higher protein levels than the constitutively expressed long form (Figure 6D). Thus, MEKK1 induction during PTI involves selection of a uORF-skipping TSS.

We finally studied the gene encoding the GATA-type TF BME3/GATA8 (At3g54810) (Figure 6F). In unchallenged seedlings, this gene produces different alternatively spliced mRNAs with long 5′-leaders, one of which contains uORFs. In contrast, as shown by CAGE and confirmed by 5′-RACE (Figure 6, F and C), transcription is induced downstream of the first intron upon PTI activation, and gives rise to an mRNA with a dramatically shortened leader. Full-length cDNAs with TSSs corresponding to the ones detected by CAGE during PTI activation have been isolated (Figure 6F), suggesting that they are both representing genuine, functional mRNAs. Although the isoform transcribed from the upstream-most TSS in unchallenged conditions splices out the clearest uORF candidates in *GATA8* (Figure 6F), it may still harbor uORF activity, because some ribosome association upstream of the major start codon was detectable in previously acquired Ribo-seq datasets (Figure 6F; Bazin et al., 2017; Yoo et al., 2020). In this case, however, the transient expression assay in *N.* *benthamiana* did not show a more efficient translation of the short isoform (Figure 6D). Thus, induction of the nearly leaderless GATA8 isoform may have significance other than affecting translation efficiency, or it may allow GATA8 induction simply because preinitiation complexes can be assembled at more than one site simultaneously.

Taken together, our results show that use of alternative transcription initiation to produce transcript isoforms differing in 5′-leader length is common during PTI activation, and that many are likely to profoundly alter translation efficiency via alternative inclusion of uORFs. It is noteworthy that none of the flg22-induced TSS changes discussed in detail here was clearly detectable by an mRNA-seq experiment conducted with standard sequencing depth (Supplemental Figure S6, see “Materials and methods”), indicating that studies of stimulus-dependent gene expression based on mRNA-seq as the sole method of transcriptome profiling miss important aspects of transcriptional reprogramming.

### Activation of eRNA-like transcription is not widespread in PTI induction

**Figure 7:**
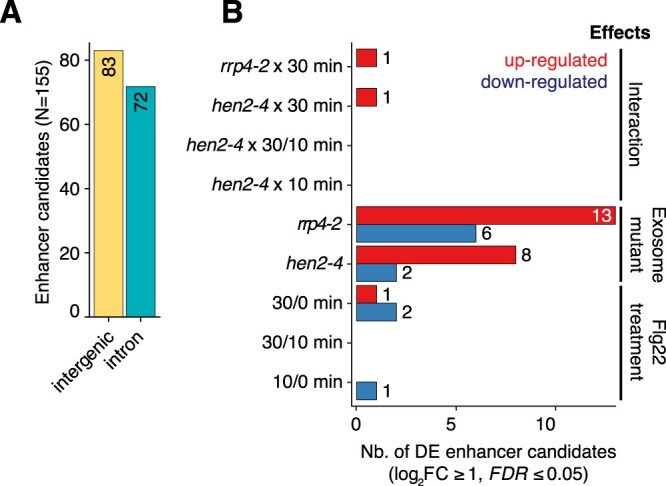
CAGE-defined enhancer candidates. A, Number of CAGE-defined enhancer candidates (*Y*-axis) in intergenic or intronic regions (*X*-axis). B, Differential expression analysis of enhancer candidates using CAGE expression. *X*-axis shows the number of enhancer candidates differentially expressed as a result of the comparisons (*Y*-axis) which are classified in three main effects: flg22 treatment (minutes), exosome-related mutant genotypes (*rrp4-2* or *hen2-4*), and the interactions thereof (e.g. *rrp4-2* x 30 min). Red and blue bars indicate upregulation and downregulated enhancer candidates, respectively.

We next addressed the question of possible existence of PTI-activated enhancers revealed by eRNA transcription. To identify such potential enhancers, we searched for short (∼500 bp) loci featuring divergent transcription in at least three samples (see “Materials and methods”). We found 155 enhancer candidates located in intergenic or intronic regions (Supplemental Data Set 8) of which 15 produced higher RNA levels in *rrp4-2* or *hen2-4* than in wt (Figure 7, A and B; Supplemental Data Set 2, see “Materials and methods”). However, only four bidirectionally transcribed loci were induced upon flg22 treatment (Figure 7B), and only a single locus showed the behavior expected from putative PTI-activated enhancers, that is, sensitivity to exosome mutation and induction by flg22 (Figure 7B). We conclude that our eRNA-focused approach did not reveal widespread existence of PTI-related enhancers.

### A rapid and transient induction of regulatory genes precedes the known PTI response

**Figure 8:**
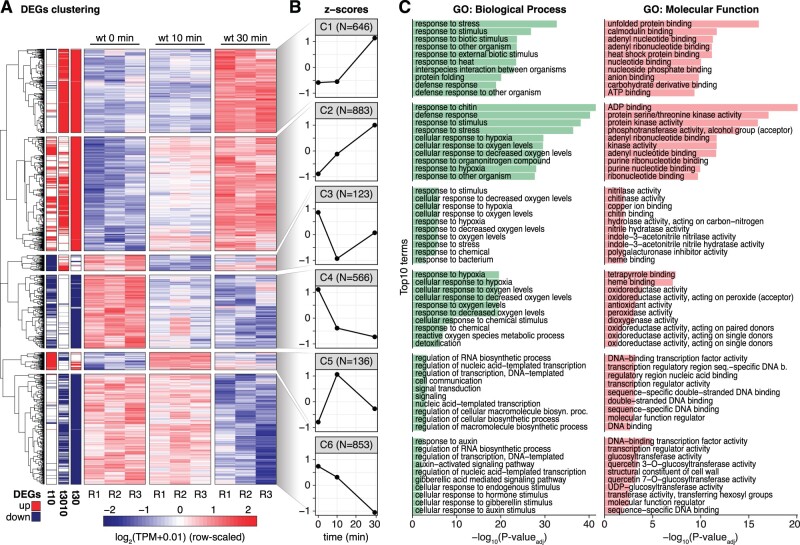
Gene expression clustering of the PTI transcriptional response. A, Heatmap of DEGs (rows) in the flg22 treatment time course. Left three columns indicate upregulation (red) or downregulation (blue) status according to the comparisons (from left to right: 10 versus 0 min, 30 versus 10 min, 30 versus 0 min). Remaining columns show wt replicates for each time point. Colors represent row-scaled, normalized CAGE expression. Vertical blocks separate the six gene expression patterns identified by hierarchical clustering. B, *Z*-score (*Y*-axis) of the average gene expression for each of the six clusters defined in (A) (C1–C6). *X*-axis shows flg22 treatment time in minutes. C, Top 10 enriched GO terms in each DEG cluster defined in (A), organized as in Figure 1C. The two independent GO categories are separated (left, biological processes; right, molecular functions).

Finally, we explored the CAGE data for patterns of gene expression change over the time course. To this end, we aggregated CAGE TCs across gene models and conducted differential expression analysis with limma (log_2_ FC ≥ 1, *FDR* ≤ 0.05, see “Materials and methods”) to identify genes responding during the flg22 treatment time course. Differentially expressed genes (DEGs) were then classified into induced or repressed sets based on hierarchical clustering of normalized gene expression values (Figure 8A; Supplemental Data Set 9). This analysis defined three clusters in each category (induced: clusters 1, 2, and 5; repressed: clusters 3, 4, and 6) according to their expression trajectory over time (Figure 8B). Cluster 1 (C1), characterized by genes activated only at 30 min after flg22 stimulation, contained the large set of previously characterized PAMP response genes, including WRKY TFs as well as defense effectors such as chitinases and other pathogenesis-related genes. Consistent with these molecular functions, the C1 gene set was enriched in the GO terms “*response to stress, biotic stimulus, and response to other organisms”* (Figure 8C, see “Materials and methods”). Genes in cluster 2 (C2) responded already after 10 min and their expression continued to increase over the 30 min time course. Thus, a sizable fraction of the known PAMP response is activated much earlier than appreciated until now, and is in temporal proximity to early signal transduction events such as MAP kinase activation. Intriguingly, cluster 5 (C5) genes were rapidly induced at 10 min, but had returned to basal expression levels at 30 min. The transient nature of C5 induction means that C5 genes may have been largely overlooked in previous profiling studies of the PAMP transcriptional response. Thus, C5 has particular potential to reveal new aspects of transcriptional reprogramming upon PAMP perception.

To further validate these central observations on the temporal nature of reprogramming of gene expression in PTI activation, we performed independent flg22 inductions and analyzed a slightly extended time course series (0, 10, 30, and 60 min after flg22 addition) by standard RNA-sequencing (RNA-seq). We found similar expression trends for the sets of genes in CAGE-defined clusters, albeit often with a temporal lag compared to the CAGE data (Supplemental Figure S7). The lag is possibly explained by the fact that CAGE detects not only mature mRNAs but also pre-mRNA species, because it relies on 5′-cap capture in combination with random-primed reverse transcription. RNA-seq, on the other hand, uses oligo-dT-selection prior to reverse transcription so that (pre-)mRNAs must have undergone 3′-end formation to be detected. This may also explain specific discrepancies between CAGE and mRNA-seq in the examples studied in detail here. For instance, although flg22 induction of the long isoform of HSFA7A is clearly detected by both CAGE and 5′-RACE at 30 min after flg22 addition, mRNA-seq failed to reveal signal in regions specific to the long isoform in the 60-min time course (Figure 3B; Supplemental Figure S6).

Taken together, our results reveal a temporal order of reprogramming of gene expression during PTI activation. Many final PAMP response genes, including regulatory factors (Supplemental Data Set 9), are induced immediately after elicitation. Intriguingly, this immediate wave of gene induction also includes transiently induced genes (C5) that we analyze in more detail below due to its outstanding potential to reveal novel aspects of PTI activation.

### Cluster 5 is enriched in TFs

**Figure 9:**
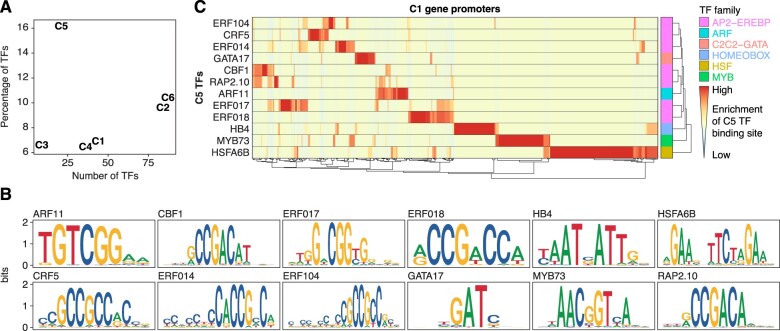
Promoters of PAMP response genes are enriched in C5 TF binding sites. A, TF genes in the DEG clusters. *X*-axis shows the number of known TFs in each of the six expression clusters from Figure 8A. The *Y*-axis shows the ratio of number of TFs to the total number of genes in each cluster. B, Sequence logos of cluster 5 TFs for which a profile could be retrieved from the JASPAR database (see “Materials and methods,” Supplemental Data Set 9). *Y*-axis shows information content in bits. C, Enrichment of binding sites from C5 TFs in the promoter regions of C1 genes. Heatmap rows indicate cluster 5 TF models, whereas columns represent C1 promoter regions. Heatmap colors show the ratio of binding site matches to the genome-wide average in all promoters (see “Materials and methods”), with red indicating high over-representation. Right-most column shows the family of the respective TF. Similar enrichment analysis for genes in all differentially expressed clusters is shown in Supplemental Figure S8.

Inspection of the functional annotation of genes contained in C5 showed that it was significantly enriched in GO terms related to TFs and nucleic acid binding activities (FDR ≤ 0.05, see “Materials and methods”) (Figure 8C). Indeed, compared to other clusters of flg22 responsive genes, the fraction of genes encoding known TFs in C5 was roughly three-fold higher (Figure 9A; see “Materials and methods”). Because of the potential of the early-induced TFs to orchestrate the eventual transcriptional output in PTI, we focused our efforts on understanding the relevance of C5 TFs.

### Links of C5 TFs to PTI signaling and establishment of immunity

We first noted that several C5 TFs have been implicated in PTI (ETHYLENE RESPONSE FACTOR104 [ERF104, At5g61600 (Bethke et al., 2009)]; ERF014 [At1g44830 (Zhang et al., 2016)]; MYB34 [At5g60890 (Frerigmann and Gigolashvili, 2014; Frerigmann et al., 2016)]) or other branches of plant immune responses to fungal and bacterial pathogens (ERF016 (At5g21960) (Zhao et al., 2021)). Strikingly, the ERF104 TF provides an example of a direct link to a major flg22-activated signal transducer, the MAP kinase MPK6. The stable MPK6-ERF104 complex dissociates within 5–15 min of flg22 perception (Bethke et al., 2009), closely matching the kinetics of MPK6 activation (Mészáros et al., 2006) and transcriptional activation of *ERF104* observed here. In addition, both knockout mutants and over-expressors of *ERF104* exhibit enhanced bacterial susceptibility (Bethke et al., 2009), pointing to the physiological relevance of the transient induction we describe here. It is also noteworthy that knockdown of ERF014 delays flg22-responsive gene expression while its overexpression is sufficient to cause bacterial resistance and hyper-responsiveness to flg22 perception (Zhang et al., 2016). These observations support the notion that an important function of at least some C5 TFs is to directly link PTI signal transducers to transcriptional reprogramming and potentiation of the immune state.

### C5 contains regulators of general stress and stem cell properties

ERF104 is also induced within minutes in response to abiotic stresses that require growth arrest (Moore et al., 2014; Vogel et al., 2014; Illgen et al., 2020), suggesting that it functions more generally in rapid stress adaptation than specifically in PTI activation. Indeed, despite their association with seemingly distinct biological processes (abiotic stress, biotic stress, and stem cell functions), a common denominator of functions of many C5 TFs may be growth restriction as a general response to stress. Clear examples of this include at least five cases: (1) the key cold stress adaptation factors CBF1 (At4g25490) and CBF2 (At4g25470) (Liu et al., 2019), also shown recently to be induced by bacterial infection (Tuang et al., 2020); (2) ERF017 (At1g19210) and ERF104, induced among other upon growth arrest-inducing intense light treatment (Vogel et al., 2014); (3) ERF018/ORA47 (At1g74930) that has direct roles in control of biosynthesis of the growth-restricting phytohormones abscisic and JA, and whose overexpression causes slow growth (Chen et al., 2016); (4) ANAC044 (At3g01600), important for arrest of cell division in response to DNA damage (Takahashi et al., 2019); and (5) heat shock TFs HSFA3 (At5g03720) and HSFA6B (At3g22830) implicated in growth restriction and induction of chaperones destined to both cytoplasm and secretory pathways (Schöffl et al., 1998; Guo et al., 2016). In this regard, we note that the uORF-skipping alternative TSSs induced in Hsp70 and the nucleotide exchange factor BAG6 (Figure 6, A and B) may be linked to the immediate induction of HSFs in C5 and C2 (HSFA4A (At4g18880), HSFB2A (At5g62020), Supplemental Data Set 9), and heat shock TFs as a group have previously been proposed to be major drivers of the growth-to-defense transition (Pajerowska-Mukhtar et al., 2012).

The vascular stem cell-specific TF WUSCHEL-LIKE HOMEOBOX 4 (WOX4, At1g46480) has not previously been associated with immunity or other stress responses, and its presence in a PAMP-induced cluster is at first glance surprising because its known functions center on stimulation of vascular stem cell proliferation (Suer et al., 2011; Etchells et al., 2013). Nonetheless, another stem cell TF, KNAT1/BP (At4g08150) was also part of C5, and the combined induction of KNAT1 and WOX4 is intriguing for two reasons. First, KNAT1 and WOX4 act redundantly to control vascular stem cell activities (Zhang et al., 2019). Second, upon auxin stimulation of root pericycle cells, this exact TF combination promotes establishment of a protective suberized, periderm layer rather than initiation of a proliferative stem cell niche destined to form a new lateral root (Xiao et al., 2020).

TFs associated with growth arrest and general stress responses were also found in C2 (e.g. HSFA4A, HSFB2A, CAMTA3 [At2g22300], CAMTA6 [At3g16940], ANAC062 [At3g49530], MYB74 [At4g05100], and several ERFs [Doherty et al., 2009; Moore et al., 2014; Vogel et al., 2014; Yang et al., 2014; Xu et al., 2015; Guo et al., 2016; Jacob et al., 2018; Illgen et al., 2020]), underscoring the multi-faceted reprogramming from growth and division to arrest immediately upon PAMP perception. Several additional TFs such as the Zinc finger/Homeobox factors HB4 (At2g44910) and HB28 (At3g50890) and the cytokinin response factor CRF5 (At2g46310; Rashotte et al., 2006) have not previously been associated with defense responses, and their inclusion in C5 therefore opens new venues to investigate their biological functions.

### Promoters of PAMP response genes are enriched in binding sites for cluster 5 TFs

We finally asked whether C5 TFs other than the experimentally verified examples discussed above (ERF104, ERF014, and MYB34) had the potential to cause transcriptional reprogramming in PTI. If this were the case, promoters of PAMP response genes should show enrichment of C5 TF binding sites. We retrieved position-specific weight matrix models describing DNA binding preferences of 12 TFs belonging to C5 from JASPAR CORE Plantae (Fornes et al., 2020) (Figure 9B; Supplemental Data Set 10, see “Materials and methods”). Using these models, we computed the enrichment compared to the genome-wide average of predicted binding sites in the promoter regions of the genes of each of clusters C1–C6 defined by differential gene expression in the flg22 time course (Figure 8A, see “Materials and methods”). Interestingly, many promoters of genes activated or repressed in PTI were indeed enriched for predicted binding sites of C5 TFs (see Figure 9C for the known PAMP response genes (C1); Supplemental Figure S8 for C1–C6). This also included C5 itself, perhaps suggesting that negative autoregulation contributes to the transient nature of its induction by flg22.

In many cases, enrichment of elements corresponding to more than one C5 TF could be identified, suggesting either a degree of functional redundancy between, or combinatorial binding of, C5 TFs in PTI. Nonetheless, we also identified many cases where only a single type of cis-element showed strong enrichment (Figure 9C). This pattern does not rule out overlapping functions of distinct TFs in regulation of PTI response genes, especially given the fact that many additional TFs (in our C2) are also induced long before the establishment of the immune state. Such a redundant setting of the system would be reminiscent of recent analyses of the transcriptional response to jasmonate in which inactivation of multiple early-induced TFs was required to observe measurable effects on the expression of later response genes (Hickman et al., 2017).

## Concluding remarks

Our study provides substantial insight into use of alternative transcription initiation sites and overall gene expression changes that take place rapidly after PAMP perception. The results should facilitate a better understanding of genetic reprogramming underlying the defense transition and establishment of the immune state in at least four ways, perhaps generalizable more widely to other types of stimulus-dependent gene expression. First, our study reveals that alternative transcription initiation is used on short time scales in different ways of considerable functional importance, including gene induction at the translational level by production of mRNA isoforms without uORFs, change in subcellular localization of encoded proteins, and dynamic expression of regulatory microproteins. Second, the discovery of very early PTI response genes facilitates the design of studies aimed at linking immediate signal transduction events such as protein kinase activation directly to changed transcriptional output. The conclusion that a very early transcriptional PTI response wave precedes the previously defined PTI response and contributes to its establishment was also reached in a thorough mRNA-seq time-course study that appeared while this work was in revision (Bjornson et al., 2021). This study of PTI induction also noted its similar pattern of establishment in distinct temporal waves across elicitation by several different PAMPs, thus allowing this conclusion on the orchestration of genetic programming in PTI induction to be generalized (Bjornson et al., 2021). It is possible that a refined temporal study to identify genuinely immediate responders, preferably through the use of nascent RNA techniques such as native elongating transcript sequencing NET-seq (Mayer and Churchman, 2016; Zhu et al., 2018; Kindgren et al., 2020), combined with existing and refined knowledge on phosphoproteome changes following PAMP perception (Rayapuram et al., 2018), will be of value to better define direct molecular links between PTI signaling and transcriptional responses. Third, the crucial, but daunting task of deciphering key elements of the texture of the TF web driving genetic reprogramming in PTI through genome-wide identification of binding sites is now tangible, because our results allow focus on a more limited number of early-responding TFs. Fourth, our study adds support to the importance of translational control in the early PAMP response (Pajerowska-Mukhtar et al., 2012; Xu et al., 2017), and hints that covalent modification of coding and/or noncoding RNAs could play roles in this regard.

## Materials and methods

### Plant materials

All *A.* *thaliana* plants are of the Col-0 ecotype. The *hen2-4* (At2g06990, SALK_091606C) mutant was described in Lange et al. (2011) and seeds were obtained from Dominique Gagliardi. The *rrp4-2* mutant is described in Hématy et al. (2016) and was kindly provided by the authors. The *fls2* (At5g46330) mutant (T-DNA insertion line SALK_062054) was obtained from the Nottingham Arabidopsis Stock Center (NASC).

### Genotyping

DNA was isolated as described in Thieffry et al. (2020). Briefly, one volume of phenol–chloroform (50:50 [v/v]) was added to freshly ground leaves in urea buffer (42% [w/v] urea, 312.5 mM of NaCl, 50 mM of Tris–HCl at pH 8, 20 mM of EDTA, and 1% [w/v] N-lauroylsarcosine). Phases were separated and DNA in the supernatant was precipitated with isopropanol and rinsed with EtOH 70% (v/v). The DNA was used as a polymerase chain reaction (PCR) template to confirm T-DNA insertion in *hen2-4* and *fls2* mutants. The point mutation in *rrp4-2* was detected by target DNA amplification and enzymatic digestion (Eco47I, *AvaII*). Genotyping primers are available in Supplemental Data Set 11.

### Growth conditions

All growth conditions were as described in Thieffry et al. (2020). Briefly, seeds were sterilized with 70% (v/v) EtOH, followed by 1.5% (w/v) sodium hypochlorite and 0.05% (w/v) Tween-20 (10 min, Sigma-Aldrich, St. Louis, MO, USA), then rinsed with sterile ddH_2_O. Clean seeds were stratified in complete darkness at 4°C for 72 h, then germinated on 1× Murashige and Skoog (MS) medium (supplemented with 1% [w/v] Suc and 0.8% [w/v] agar) under sterile conditions (Petri-dishes) with long-day light cycles (16-h light/8-h dark photoperiod, 130-µmol photons m^−2^s^−1^ at 21°C, cat. No. Master TL-D 36W/840 bulbs; Philips). Intact 12-day-old seedlings were transferred to 8 mL of 1× liquid MS medium (as above) in 6-well plates (Nunc, cat. No. 140675) and acclimated for 2 days with mild agitation (130 rpm), under identical light and temperature settings as above.

### Flagellin treatments

Flg22 peptide with sequence Ac-QRLSTGSRINSAKDDAAGLQIA-OH was obtained from Schafer-N (www.schafer-n.com) with purity >95%. Flg22 peptide was dissolved in dimethyl sulfoxide (DMSO) at 1 mg/mL. Biological replicates consisting of a pool of 10 seedlings were subjected to 3.3 µM of flg22 and 0.77% of DMSO under constant agitation (130 rpm) for 10 and 30 min for the CAGE samples, and 0, 10, 30, and 60 min for the RNA-seq samples. Seedlings were removed from the media and immediately flash-frozen in liquid nitrogen before undergoing RNA extraction.

### Definition of biological replicates

Biological replicates were produced in the following way: seeds from the same seed batch were germinated on different 1× MS agar plates as described above, but grown in parallel and with plates next to one another in the same growth cabinet. Similarly, flg22 inductions were carried out separately for each replicate, with each replicate in a distinct well, but were done at the same time under the same growth conditions.

### Total RNA extractions

Total RNA was extracted as described in Thieffry et al. (2020), with the addition of samples treated with flg22 peptide for 10, 30, and 60 min. Briefly, plant material was flash-frozen and 1 mL of TRI-Reagent (Sigma-Aldrich) was added to 100 mg of finely ground tissue. Following chloroform phase separation, the aqueous phase was transferred to a fresh tube and the RNA was precipitated with one volume of isopropyl alcohol (400 µL) for 30 min at room temperature. Total RNA was pelleted by centrifugation (10 min at 15,000 rpm and 4°C), rinsed with 70% (v/v) EtOH and re-suspended in RNAse-free ddH_2_O. A further polysaccharide precipitation was conducted as described by Asif et al. (2012) to remove contaminants and obtain higher quality RNA material. All RNA samples were assessed for concentration and purity using the NanoDrop ND-1000 (Thermo Fisher Scientific, Waltham, MA, USA) and absence of RNA degradation was confirmed on a Bioanalyzer 2100 with High Sensitivity RNA chip (RNA 5000 Pico; Agilent Technologies, Santa Clara, CA, USA).

### Reverse transcription-quantitative polymerase chain reaction

Initial validation of the flg22 treatment was assessed with reverse transcription-quantitative PCR (RT-qPCR) on a QuantStudio version 6 instrument (Thermo Fisher Scientific), using the total RNA extracted for the CAGE library preparations and the ΔΔ*C_t_* method. The list of exon/exon junction spanning primers is available in Supplemental Data Set 11.

### CAGE library construction, filtering, and mapping

CAGE libraries were prepared as in Takahashi et al. (2012) from 5 µg of total RNA and sequenced on an Illumina HiSeq 2000 platform with 30% of Phi-X spike-ins. Filtering and mapping of CAGE libraries were processed as in Thieffry et al. (2020). Briefly, linker sequences were trimmed with FASTX Toolkit version 0.0.13 (http://hannonlab.cshl.edu), and only the first 25 nt were retained. Filtering for a minimum Phred (Phil’s Read Editor) score of Q30 in 50% of the bases was applied. Clean reads were mapped on TAIR10 with Bowtie version 1.1.2 (Langmead et al., 2009) and the 5′-ends of uniquely mapped reads were summed at single base pair resolution to obtain CAGE TSSs (CTSSs). CTSS coordinates were offset by 1 bp to account for the G-addition bias (Carninci et al., 2006).

### RNA-seq library construction and analysis

Purified RNA from samples that underwent 0, 10, 30, and 60 min of flg22 treatment were sent to Novogene, Hong Kong for preparation of polyDT-selected, unstranded, paired-end 150 bp library and sequencing on a Novaseq 6000 platform. Basecalling was conducted with Cassava (version 1.8). Adapters were trimmed with Cutadapt version 1.18 (Martin, 2011) and clean reads were mapped on the TAIR10 reference genome with HISAT2 (Kim et al., 2019) using default parameters and keeping only concordant paired alignments. Salmon (Patro et al., 2017) was used for transcript and gene quantification and digital counts were normalized to reads per kilobase million.

### Ribo-seq analyses

Ribo-seq datasets from Hsu et al. (2016), Xu et al. (2017), Bazin et al. (2017), and Yoo et al. (2020) were downloaded from the Gene Expression Omnibus (GEO). Following adapter removal with Cutadapt version 1.18 (Martin, 2011), clean reads were mapped on the TAIR10 reference genome using Bowtie (version 2.3.4.3) in local mode with default parameters. SAM alignment files were transformed into bigWig format and TPM-normalized. Full details of the Ribo-seq datasets are available in Supplemental Data Set 3.

### 5′-RNA ligase-mediated RACE

5′-ends of selected RNAs were isolated according to the instructions from the GeneRacer kit (Invitrogen, Waltham, MA, USA), with minor modifications. Briefly, 2 µg of total RNA was dephosphorylated (CIAP, Invitrogen), decapped (mRNA Decapping Enzyme, NEB Ipswich, MA USA) and ligated to the GeneRacer RNA adaptor (Integrated DNA Technologies) using T4 RNA ligase (Invitrogen). Ligated RNAs were reverse transcribed using RevertAid First Strand cDNA Synthesis kit (Invitrogen) and a reverse Gene-specific Primer (GSP). Next, 30 cycles of PCR were run with the GeneRacer 5′ primer and the GSP. Then, 0.5 µL of these reactions was used as a template for 30 cycles of nested PCR using the GeneRacer 5′-nested primer and a reverse GSP nested primer. Finally, 10 µL of the nested PCRs were loaded, separated, and visualized by ethidium bromide staining in a 1% agarose gel. The full list of RNA and DNA oligonucleotides used is listed in Supplemental Data Set 11.

### Cloning procedures

All DNA fragments were PCR amplified using Phusion High-Fidelity DNA Polymerase (New England Biolabs) and gel-purified with GeneJET gel extraction kit (ThermoFisher Scientific, Waltham, MA, USA). DNA fragments were cloned into pENTR/D (ThermoFisher Scientific). Sequences of all the recombinant plasmids were confirmed by Sanger sequencing. DNA fragments were subcloned into binary vectors (see below) using Gateway LR Clonase II Enzyme mix (Invitrogen). For cloning of the short- and long isoforms of GATA8, HPS70b, MEKK1, BAG6 (uORFs) and the long isoform of HSFA7A, cDNAs were prepared from mock or flg22-treated Arabidopsis seedlings with the RevertAid First-Strand cDNA Synthesis Kit (ThermoFisher Scientific) following the manufacturer’s instructions and using GSPs. LR reactions were used for subcloning all the isoforms into pGWB514 (C-terminal fusion with 3xHA). For cloning of PUS5 short- and long isoforms, DNA fragments were PCR amplified using Arabidopsis genomic DNA as a template. Both isoforms were subcloned into the pGWB505 binary vector to generate C-terminal fusions with eGFP. All primers used for cloning procedures are detailed in Supplemental Data Set 11.

### Construction and analysis of transgenic Arabidopsis lines

Arabidopsis transgenic lines were generated by transformation with *Agrobacterium tumefaciens* carrying the binary vectors by the standard floral dipping method (Clough and Bent, 1998). Transformants plants were selected into MS plates supplemented with hygromycin (40 μg mL^−1^). Plants were grown at 21°C with a photoperiod of 16-h light/8-h dark (long-day conditions). Presence of the recombinant protein (long HSFA7A-HA) was confirmed by immunoblot (Supplemental Figure S3).

### Transient expression in *N. benthamiana*

Transient expression assays in *N. benthamiana* were performed as described in Rufián et al. (2015) with minor modifications. Briefly, 2- to 3-week-old plants were inoculated with a solution containing 10- mM MES (Sigma), 10-mM MgCl_2_, 100-μM 3′,5′-dimethoxy-4′-hydroxyacetophenone (acetosyringone; Sigma), and the desired *Agrobacterium* carrying the binary vector (OD_600_ = 0.5). Samples were taken at 2 days post infiltration and analyzed by confocal microscopy or immunoblot.

### Confocal microscopy

*Nicotiana* *benthamiana* leaf discs transiently transformed with either *PUS5*(short)-*GFP* or *PUS5*(long)-*GFP* constructs were imaged with a Zeiss LSM700 confocal microscope. The GFP fluorophore was excited with a 488-nm laser, and emitted fluorescence was captured using the filter configuration pre-set for GFP in the Zeiss microscope software.

### Immunoblotting

Leaf tissue was frozen in liquid nitrogen and mechanically disrupted directly in Laemmli buffer (70 mM Tris–HCl pH 6.8, 10% glycerol, 100-mM DTT, 1% LDS, and 0.01% BPB), boiled 5 min and centrifuged 1 min at 14,000 rpm to remove tissue debris. For each experiment, fresh tissue was weighed prior to freezing to ensure that the same amounts were analyzed for each sample in the series. Proteins were separated by SDS–PAGE and transferred onto a nitrocellulose membrane (Amersham, GE Healthcare Life Science). Blocking was performed in a solution containing 1× PBS buffer, 5% skimmed milk, and 0.05% Tween-20 for 30 min at room temperature. Membranes were incubated with the primary antibody (anti-HA 12CA5, Sigma, 1:4,000 dilution, cat. no. 11583816001) at 4°C overnight. Membranes were washed three times with 1× PBS, 0.05% Tween-20 buffer and incubated with the (HRP)-conjugated secondary antibody (cat. no. A0168, Sigma) for 1 h at room temperature. HRP activity was detected by enhanced chemiluminescence.

### RNA gel blot

Ten micrograms of total RNA extracted from *N. benthamiana* leaves were dissolved in loading buffer (1× HEPES [20-mM HEPES, 1-mM EDTA, and 17-mM KOH, pH 7.8], 45% formamide, 16% formaldehyde, 16% ethidium bromide, and bromophenol blue) and denatured by incubation for 5 min at 95°C. RNA was run in a 1% agarose gel prepared with 16% formaldehyde 1× HEPES buffer for 3 h and transferred onto a nylon membrane (Amersham Hybond-NX, GE Healthcare Life Sciences) by capillary flow in the presence of 20× SSC buffer (3-M NaCl and 300-mM sodium citrate, pH 7.0). After UV-crosslinking (Stratalinker UV 1800, Stratagene), membranes were incubated in PerfectHyb Plus Hybridization buffer (Sigma-Aldrich) at 42°C for 1 h. A ^32^P-radio-labeled probe was synthesized using the Prime-a-Gene kit (Promega, Madison, WI, USA) with an HA-NOST PCR fragment as template (see Supplemental Data Set 11 for primer sequences), and hybridized to membranes overnight at 42°C with gentle rotation in the same buffer. After hybridization, membranes were washed with 2× SSC, 2% SDS at 42°C (three washes of 10 min). Signal was detected by exposure to a phosphoimager screen (TyphoonTM FLA 7000, GE Healthcare Life Science).

### Analysis of CAGE tag clusters

Most of CAGE data analyses were conducted with the CAGEfightR version 1.2 package (Thodberg et al., 2019), as described in Thieffry et al. (2020). CTSSs (genomic 5′-end positions supported by CAGE tags) with at least one count in a minimum of three libraries were retained. CAGE TCs were generated by neighbor-clustering of CTSSs from the same strand with a maximum distance of 20 bp. Following quantification, CAGE TCs were further filtered for a minimum of 1 TPM in at least three libraries (the smallest group size in our experiment). Position of the highest signal within a TC defined the TC peak. Principal component analysis was conducted on CAGE TCs using the scaled and centered TPM-normalized expression across libraries. CAGE TCs were annotated on the basis of the position of their peak signal against a hierarchical annotation (see Figure 2B, right) constructed from TAIR10 (TxDb.Athaliana.BioMart.plantsmart28, Bioconductor). Analysis of fractional CAGE TC usage within genes was conducted as in Xu et al. (2019), where TCs in a multi-TC gene were assigned a rank according to the degree of their contribution to their cognate gene total expression (rank 1: most contributing TC, rank 2: second most contributing TC, and so forth). Only ranks 1–4 were shown. Simpson index of CAGE TC diversity within genes was computed with the *diversity* function from the *vegan* R package (https://github.com/vegandevs/vegan). Enhancer candidates were identified with the *clusterBidirectionally* function from CAGEfightR, using a window size of 500 bp and balance threshold of 0.95 (Bhattacharyya coefficient). Further requirements for the final set of enhancer candidates were (1) a bidirectional CAGE signal existing in three or more samples, (2) overlapping with TAIR10 intergenic or intronic regions based on enhancer candidate midpoint, and (3) found in nuclear chromosomes (Chr I–V).

### GO enrichment

Gene set enrichment analyses were conducted with the gProfileR package (Reimand et al., 2007), using all detected genes as background, and correcting resulting *P*-values for multiple testing with the Benjamini–Hochberg method (Benjamini et al., 2001).

### Alternative TSSs and differential TC usage

All analyses relative to alternative TSSs used intragenic CAGE TCs contributing at least 10% to the total expression of their host gene in at least three libraries. Cases of DTU in the flg22 time course were determined with the *diffSplice*/*topSplice* functions from the limma package (Ritchie et al., 2015), testing for a difference of log_2_(FC) of each TC compared to the average log_2_(FC) of all other TCs within the same gene (*t* test). A minimum effect size of 2 in at least one time course comparison was required, and *P*-values were corrected for multiple testing with the Benjamini–Hochberg method (Benjamini et al., 2001).

### Disruption of protein domains

The catalog of protein domains for *A.* *thaliana* was downloaded from TAIR10 official website (www.arabidopsis.org). The positions of amino acids were mapped back to the reference genome with the *pmapFromTranscripts* function from the GenomicFeatures Bioconductor package (Lawrence et al., 2013). Sense CAGE TCs falling within a protein domain or located downstream of a domain within the same gene were defined as domain disruptive, using TC peak as reference position.

### Target peptide analysis

Amino acid sequences of all TAIR10 proteins were retrieved from www.arabidopsis.org and scanned with SignalP version 5.0 and TargetP version 2.0 (Almagro Armenteros et al., 2019) to identify localization signals in the 5′-end. Position of the predicted cleavage site was multiplied by three and mapped back in genomic space. First, loss of localization signal was assessed by considering CAGE TC peaks located within or downstream predicted signal peptides. In a second time, identification of relative TSS switches leading to inclusion or exclusion of a signal peptide was considered on the basis of detected DTUs (see above).

### uORF analysis

The set of *A.* *thaliana* uORFs was obtained from Kurihara et al. (2018). After duplication and removal of inconsistent entries, uORF regions were mapped into genomic space. CAGE TCs within or downstream of uORFs were selected on the basis of their TC peak location. The only exception to this procedure were the uORFs in the *MEKK1* gene which were not contained in the Kurihara et al. (2018) dataset and which were identified by manual sequence scanning.

### Differential expression analyses and clustering

Differential expression analysis was carried out with the *edgeR* (Robinson et al., 2010; McCarthy et al., 2012) and *limma* (Ritchie et al., 2015) Bioconductor packages. Counts were modeled using ∼ genotype * timepoint, to capture the effects of the exosome mutants (*genotype*), the time course (*timepoint)*, and their interaction. The same analysis was executed at gene level, where TC counts were aggregated when belonging to the same gene. DEGs in the flg22 induction were selected for hierarchical clustering (with clustering method: *complete*, and clustering distance: *correlation*) on the basis of the normalized expression from the wt samples, and visualized with the *pheatmap* R package (https://github.com/raivokolde/pheatmap).

### TFs and enrichment of binding sites

AGRIS AtTFDB (Palaniswamy et al., 2006) database was used to identify genes encoding TFs in our experiment on the basis of Arabidopsis Genome Initiative gene identification numbers. The position weight matrices (PWM) of each TF-encoding gene found in the flg22-responsive cluster 5 was recovered from JASPAR Plantae CORE database (Fornes et al., 2020). When no specific PWM was available, the protein sequence of the TF was used for JASPAR profile inference. A list of matrix identification numbers is available in Supplemental Data Set 9. Promoter regions, defined as the 500-bp stretch upstream of the closest CAGE TC peak to each annotated TAIR10 TSS, were scanned for TF motif matches with Find Individual Motif Occurrences (Grant et al., 2011), requiring a minimum similarity of 70%. Finally, for each TF in cluster 5, the genome-wide average number of matches within promoters was used as the background frequency to obtain the enrichment ratio in promoters of each DEG cluster. The resulting binding enrichments were visualized as hierarchically clustered heatmaps.

### Accession numbers

CAGE libraries and processed files are available on the GEO database (www.ncbi.nlm.nih.gov/geo) under accessions GSE136356 and GSE143590. RNA-seq libraries and processed files are available under accession GSE144356. CAGE TCs and CAGE signals for all libraries are available on the online JBrowse of www.arabidopsis.org, as well as R scripts via GitHub: https://github.com/athieffry/Thieffry_et_al_2021.

## Supplemental data 

The following materials are available in the online version of this article.

**Supplemental Figure S1.** PTI response marker genes in flg22 induction and DEGs (supports Figure 1).

**Supplemental Figure S2.** CAGE signal in *hen2-4* mutant for our examples of alternative TSS usage (supports Figures 3–6).

**Supplemental Figure S3.** Detection of the constitutively expressed HSFA7A-3xHA long isoform in Arabidopsis transgenic lines (supports Figure 3D).

**Supplemental Figure S4.** Subcellular localization of short and long forms of PUS5-GFP (supports Figure 5, extended version of Figure 5C).

**Supplemental Figure S5.** Complete immunoblots and RNA gel blots for GATA8, BAG6, HSP70b, and MEKK1 (supports Figure 6D).

**Supplemental Figure S6.** mRNA-seq signal for examples of alternative TSS usage discussed in detail (supports Figures 3–6).

**Supplemental Figure S7.** Validation of CAGE-defined expression clusters by RNA-seq (supports Figure 8).

**Supplemental Figure S8.** C5 TF binding sites in promoters of DEGs belonging to all clusters in the flg22 time course (supports Figure 9).

**Supplemental Data Set 1.** CAGE TCs.

**Supplemental Data Set 2.** Differential expression analyses.

**Supplemental Data Set 3.** Ribo-seq datasets.

**Supplemental Data Set 4.** CAGE TC switches.

**Supplemental Data Set 5.** Protein domains.

**Supplemental Data Set 6.** Localization signals.

**Supplemental Data Set 7.** uORFs.

**Supplemental Data Set 8.** Enhancer candidates.

**Supplemental Data Set 9.** DEG clusters.

**Supplemental Data Set 10.** JASPAR matrices.

**Supplemental Data Set 11.** Oligonucleotides.

## Supplementary Material

koac108_Supplementary_Data

## Notes

These authors contributed equally (A.T. and D.L.M).

A.T., A.S., and P.B. designed the study. J.B. made the CAGE libraries. A.B. helped with total RNA extractions. D.L.M. conducted 5′-RACE experiments, constructed and analyzed HSFA7A transgenic lines, designed, and constructed plasmids for transient expression, and carried out transient expressions and subsequent molecular analyses. M.G.M. assisted with construction of plasmids for transient expression. S.B. and D.L.M. did confocal microscopy analysis. A.T. conducted all other experiments and computational analyses. A.T. and D.L.M. produced the figures. P.B., A.T., D.L.M., and A.S. wrote the paper with inputs from all authors.

The author responsible for distribution of materials integral to the findings presented in this article in accordance with the policy described in the Instructions for Authors (https://academic.oup.com/plcell) is: Peter Brodersen (pbrodersen@bio.ku.dk).
