# Supplementary material for: PAMP-triggered genetic reprogramming involves widespread alternative transcription initiation and an immediate transcription factor wave
Source: Plant Cell. 2022 Apr 11;34(7):2615–37. doi: 10.1093/plcell/koac108 (PMC9252474; doi:10.1093/plcell/koac108)
Supplement: koac108_Supplementary_Data [file koac108_supplementary_data.zip › koac108-suppl_data/tpc.22.00124Supplemental Figures.pdf]

Supplemental Figure S1

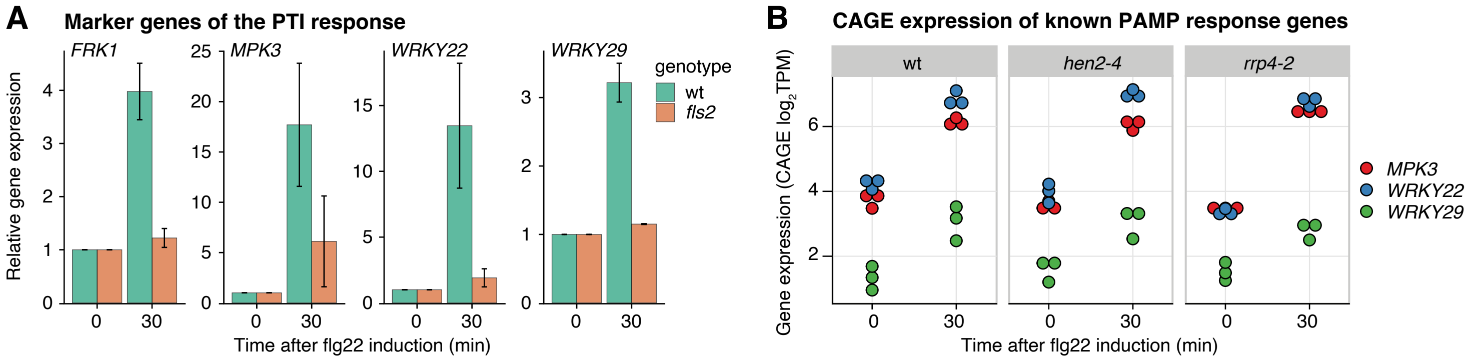

Supplemental Figure S1. PTI response marker genes in flg22 induction and DEGs (supports Figure 1).
(A) Relative expression of *FRK1* (At2g19190), *MPK3* (At3g45640), *WRKY22* (At4g01250), and *WRKY29* (At4g23550) as measured by real-time polymerase chain reaction at 0 and 30 minutes after flg22 treatment (X-axis). The Y-axis shows expression relative to time point zero. Colors indicate genetic backgrounds and error bars indicate standard deviation.
(B) CAGE expression of PAMP-response genes. X-axis shows time after flg22-induction, in minutes. Y-axis is the normalized CAGE expression in transcripts per millions (TPM), on log_2 scale. Colors indicate genes and panels separate genotypes.

Supplemental Figure S2

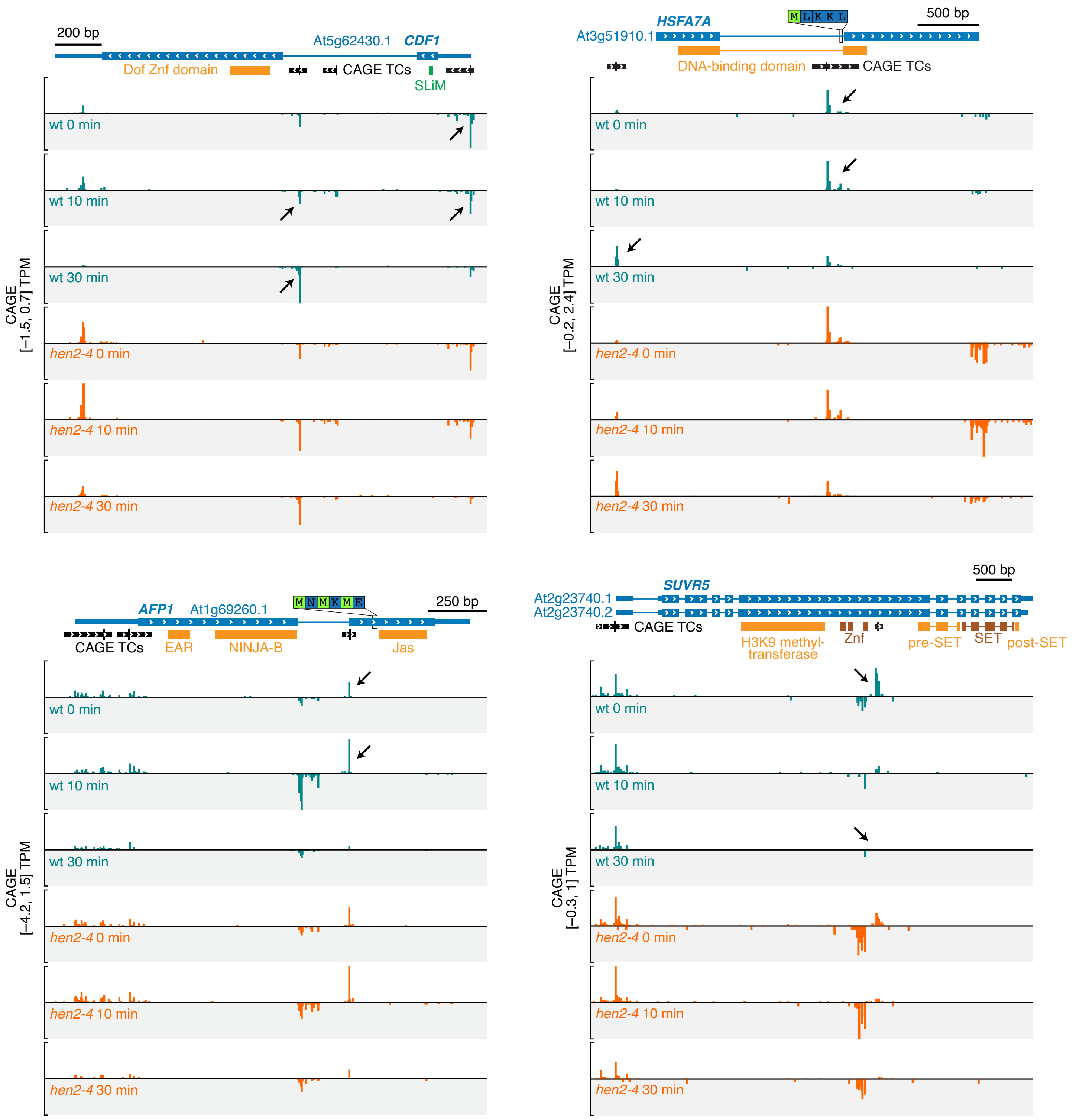

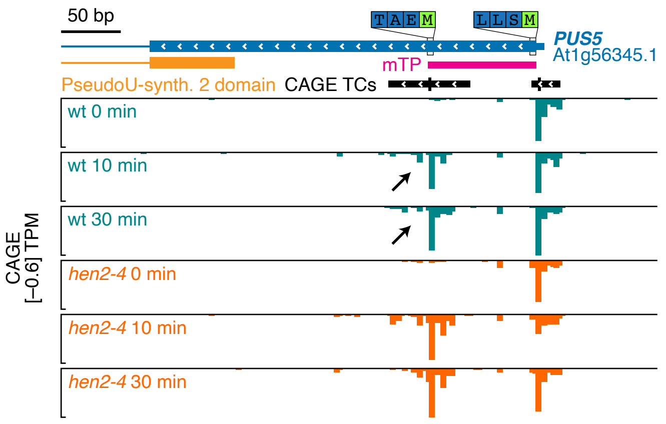

Supplemental Figure S2. CAGE signal in *hen2-4* mutant for our examples of alternative TSS usage in flg22-induced PTI (supports Figures 3-6). Genome browser views of *CDF1*, *HSFA7A*, *AFP1*, *SUVR5*, and *PUS5* showing CAGE signal tracks for wild type and *hen2-4* mutant at 0, 10, and 30 minutes of flg22 treatment. Organized as in **Figure 3A**.

Supplemental Figure S2 (continued)

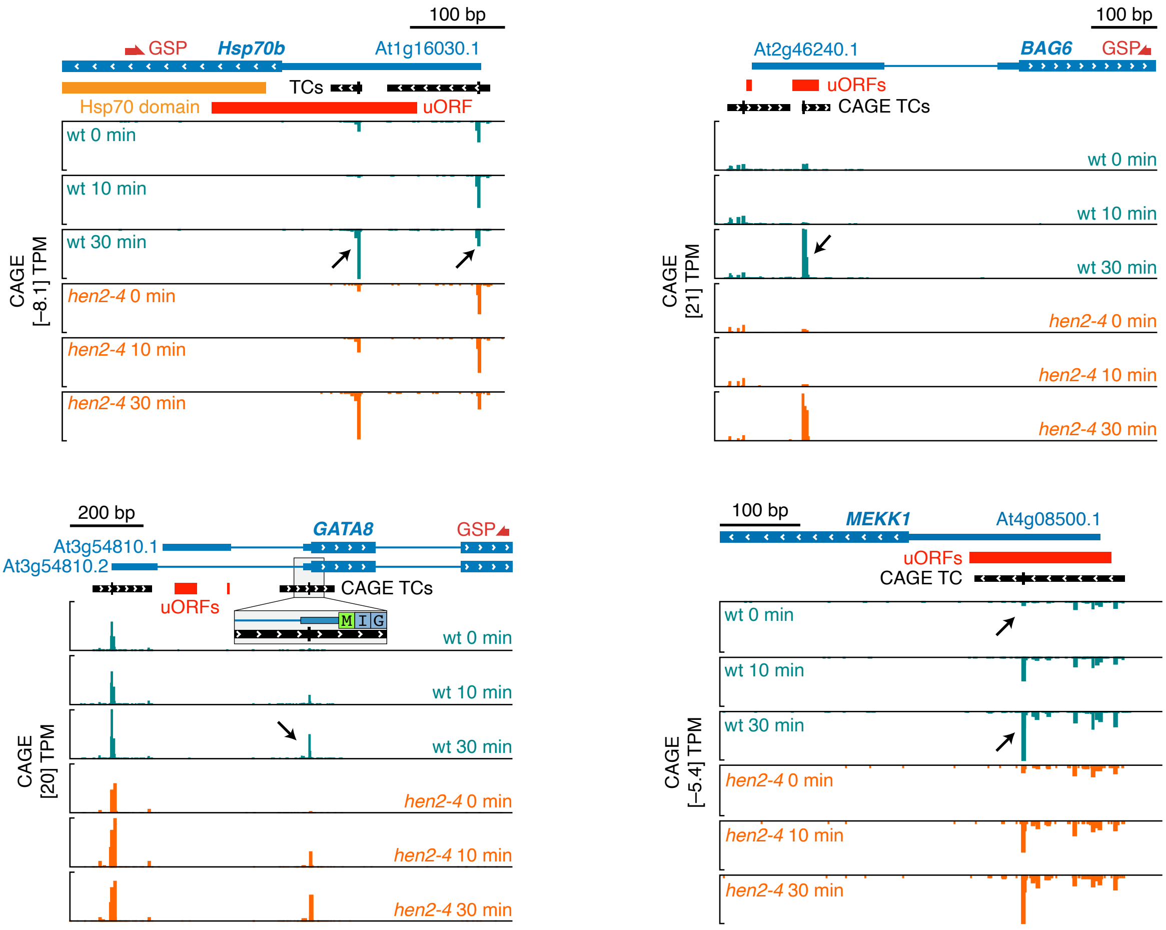

Supplemental Figure S3

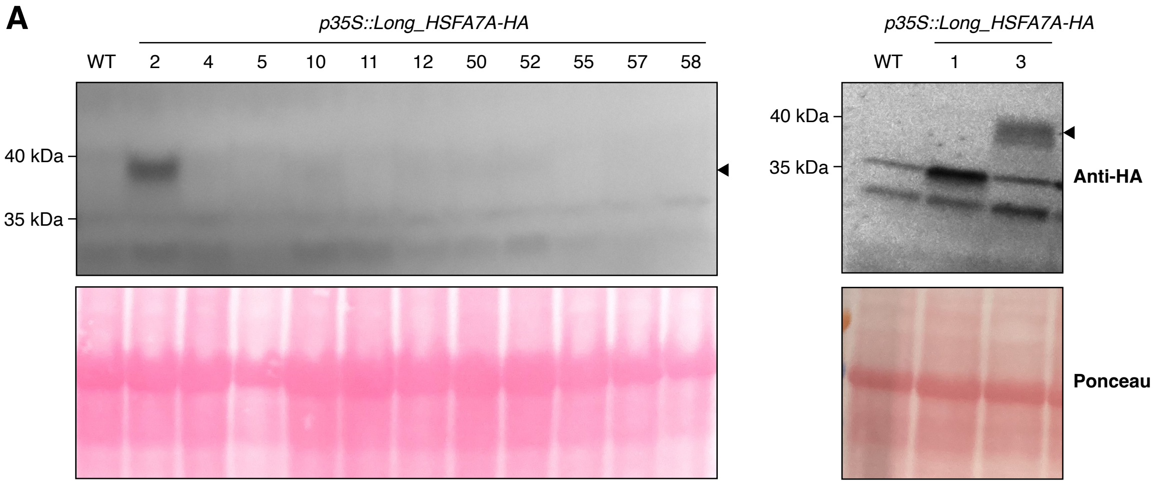

Supplemental Figure S3. Detection of the constitutively expressed *HSFA7A-3xHA* long isoform in Arabidopsis transgenic lines (supports Figure 3D).
Immunoblot of protein extracts prepared from independent transgenic lines transformed with the *p35S-HSFA7A(long)-3xHA* construct. The two lines (#2 and #3) showing detectable *HSFA7A-3xHA* expression were the only ones showing the slow growth phenotype giving rise to small rosettes, as shown in **Figure 3D**.

Supplemental Figure S4

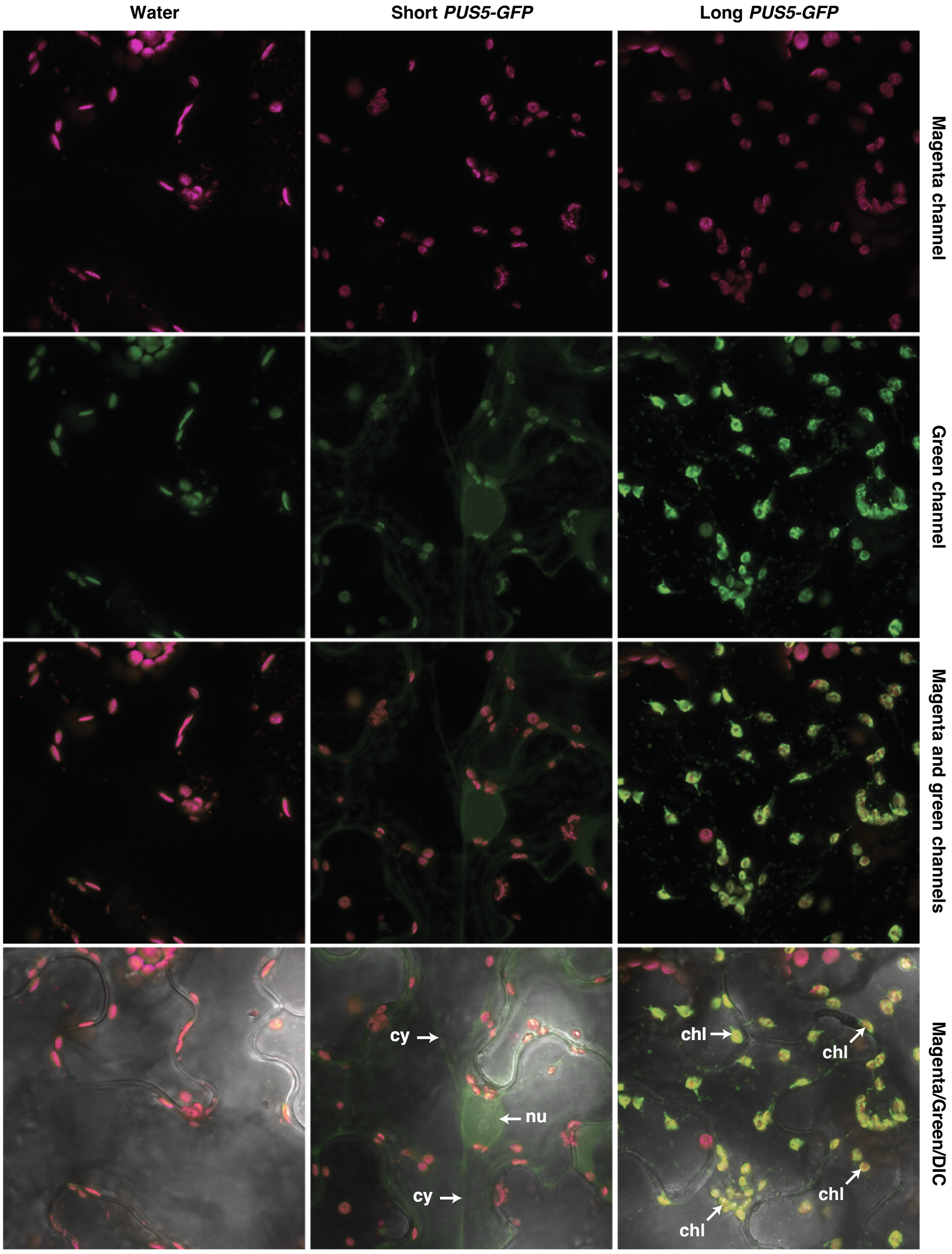

Supplemental Figure S4. Subcellular localization of short and long forms of PUS5-GFP (supports Figure 5, extended version of Figure 5C).
Confocal microscopy images of *Nicotiana benthamiana* leaf discs infiltrated with either water (mock, negative control), or with *Agrobacterium* harboring binary plasmids with short or long isoforms of *PUS5* fused to *GFP*. Each column shown the same leaf area imaged in different ways. First row (from the top): magenta channel showing chlorophyll autofluorescence, thereby identifying chloroplasts. Second row: green channel showing both GFP and (weak) green autofluorescence. Autofluorescence can be identified as green signal appearing in the mock-treated negative control. It is to be noted that (i) we did not find one instance of green signal appearing in the nucleus or cytoplasm in mock-treated leaf discs, (ii) the green signal in chloroplasts obtained the long form of PUS5-GFP was substantially more intense than that obtained in mock-treated leaf discs. Third row: overlay of signal obtained in magenta and green channels. Fourth row: an overlay of magenta/green channels on a differential interference contrast (DIC) microscopy image of the same area. Nu, nucleus; cy, cytoplasm; chl, chloroplast.

Supplemental Figure S5

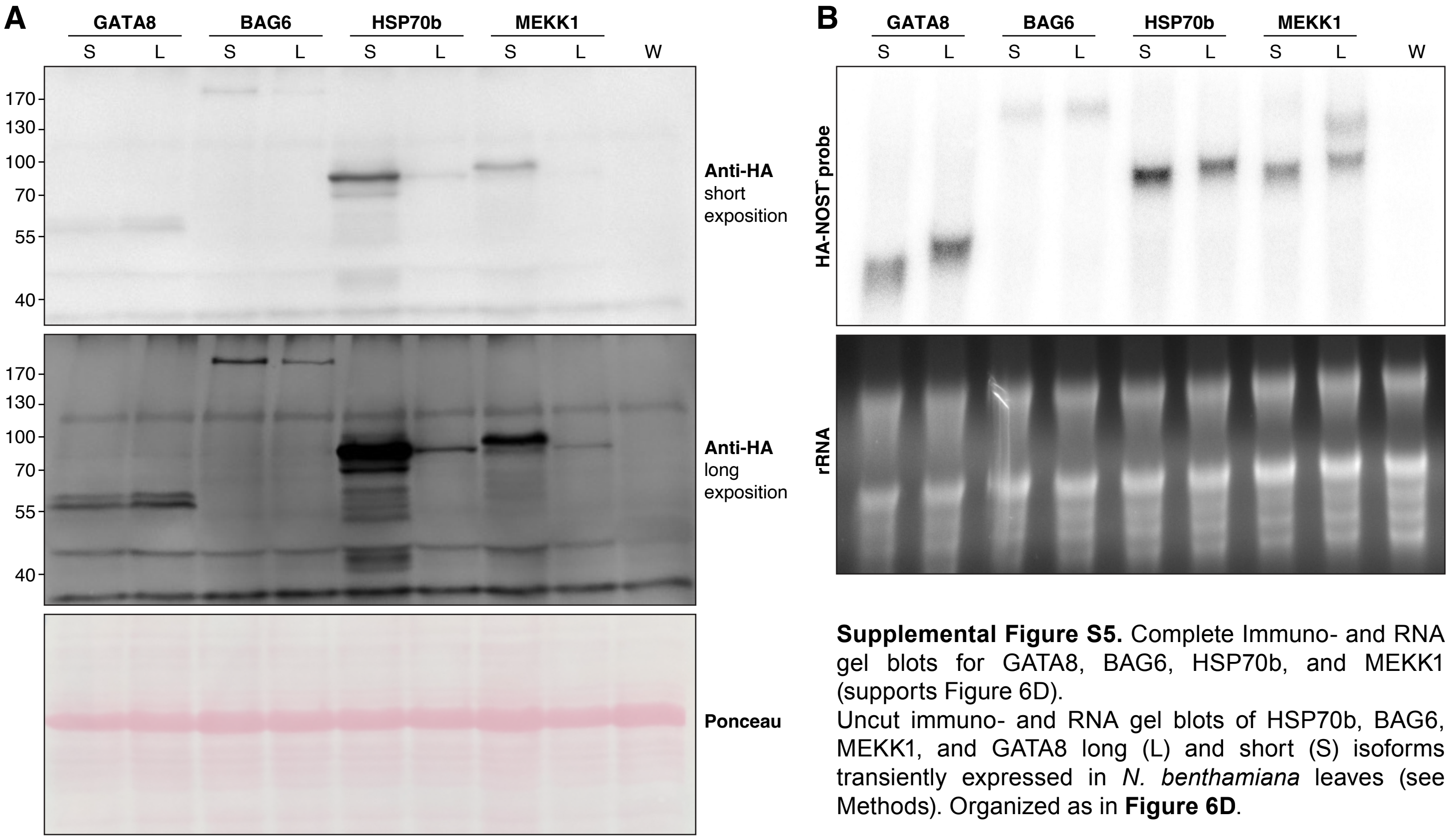

Supplemental Figure S5. Complete Immuno- and RNA gel blots for GATA8, BAG6, HSP70b, and MEKK1 (supports Figure 6D).

Uncut immuno- and RNA gel blots of HSP70b, BAG6, MEKK1, and GATA8 long (L) and short (S) isoforms transiently expressed in *N. benthamiana* leaves (see Methods). Organized as in **Figure 6D**.

Supplemental Figure S6

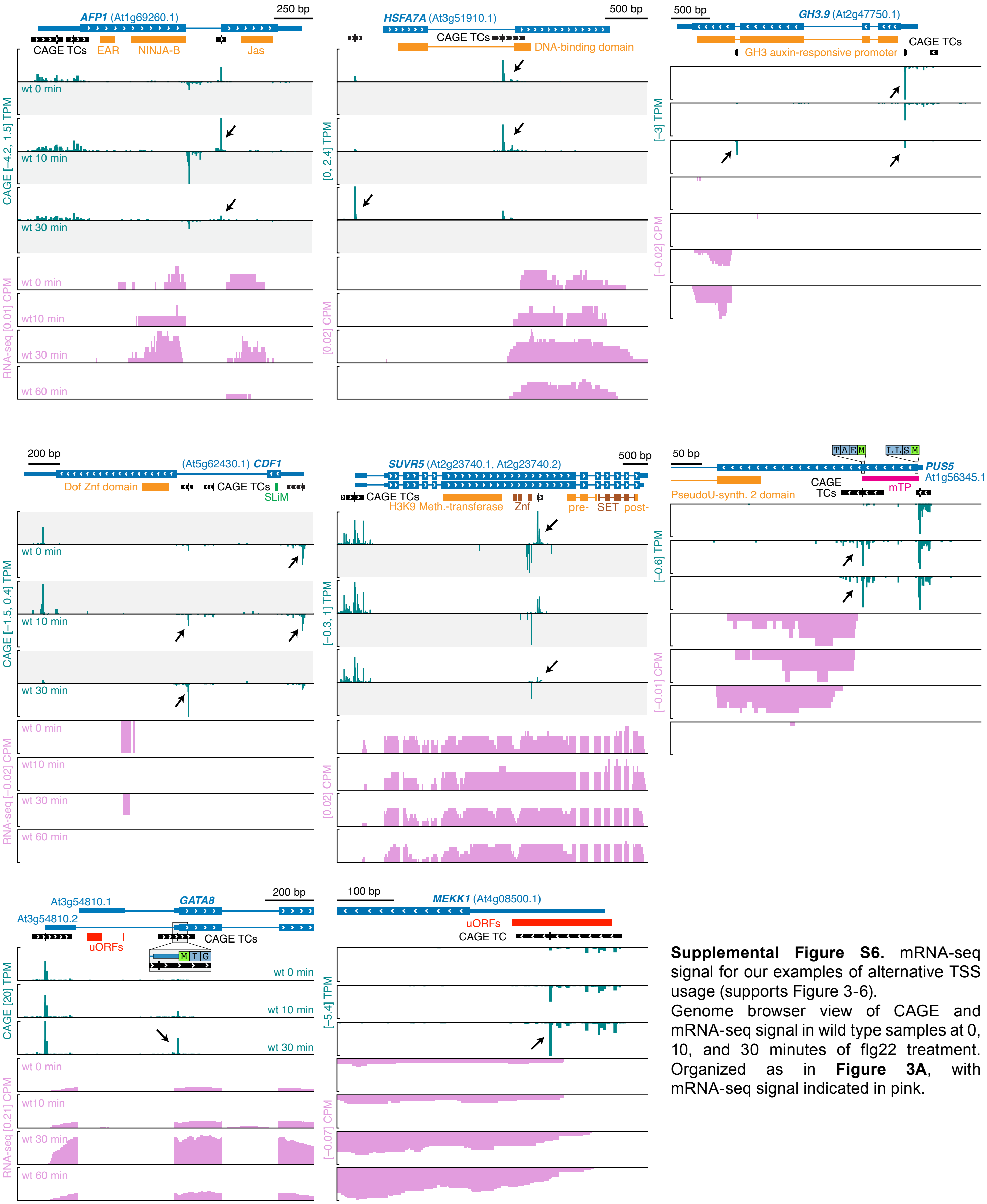

Supplemental Figure S6. mRNA-seq signal for our examples of alternative TSS usage (supports Figure 3-6).
Genome browser view of CAGE and mRNA-seq signal in wild type samples at 0, 10, and 30 minutes of flg22 treatment. Organized as in **Figure 3A**, with mRNA-seq signal indicated in pink.

Supplemental Figure S7

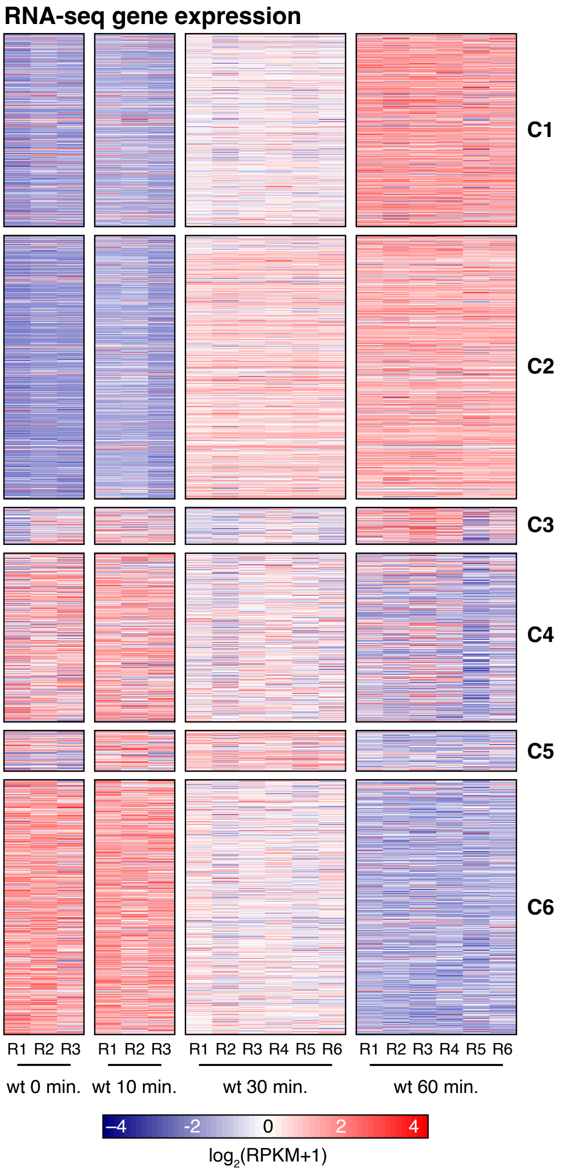

Supplemental Figure S7. Validation of CAGE-defined gene expression clusters by mRNA-seq (supports Figure 8).
Heatmap of gene expression for wild type samples treated with flg22 peptide for 0, 10, 30, and 60 minutes, as measured by RNA sequencing. Rows represent genes. Vertical ordering of genes and clusters are defined by the clustering of CAGE data in **Figure 8A**. Columns show the replicates at the indicated time points. Colors reflect log-transformed RPKM expression with a pseudocount of 1. C1-C6 labels to the right refer to CAGE-defined DEG clusters from **Figure 8A**.

Supplemental Figure S8

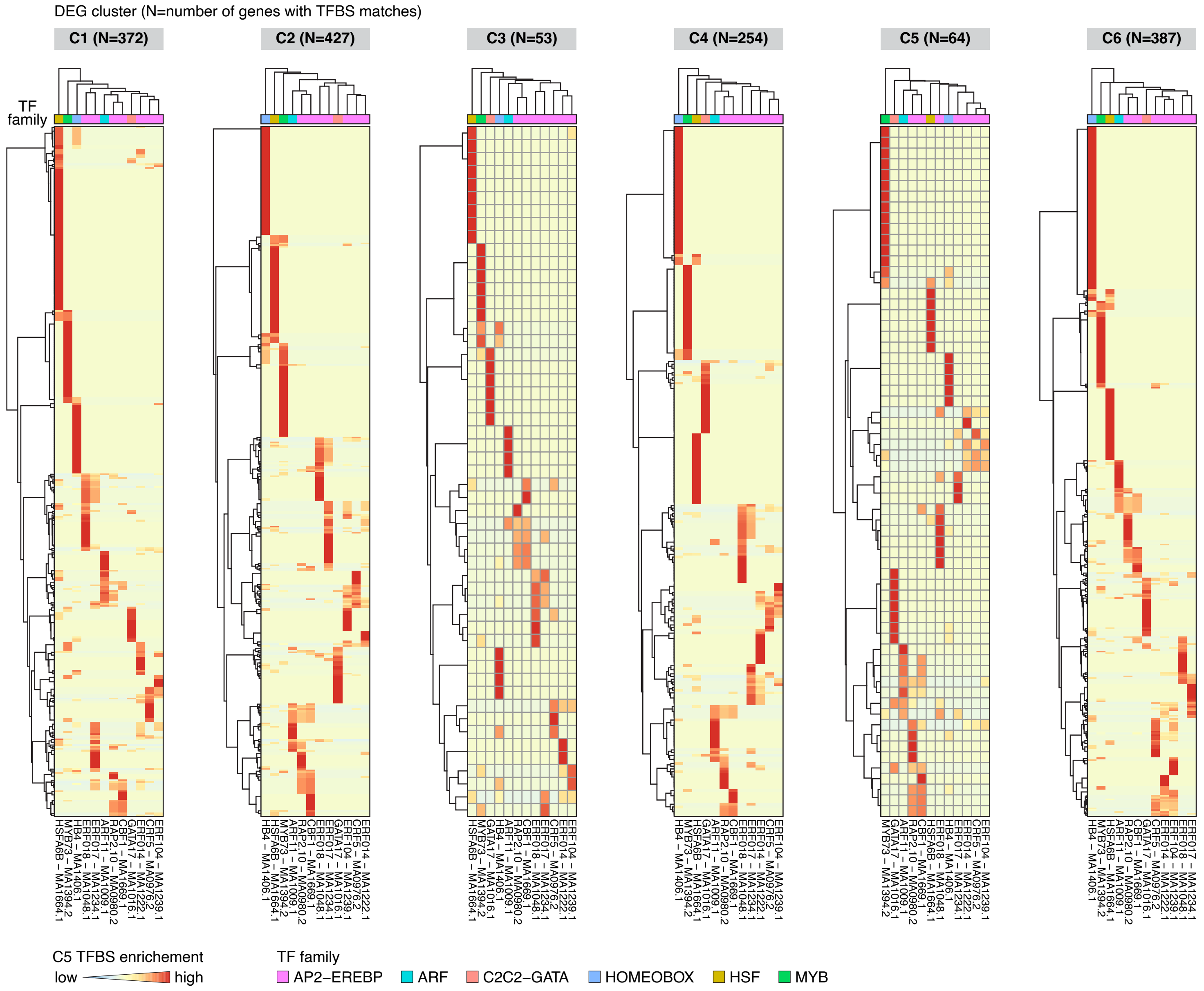

Supplemental Figure S8. C5 transcription factors binding sites in promoter of flg22 time course cluster DEGs (supports Figure 9). Enrichment of cluster 5 transcription factor binding sites in the promoter regions of genes responding to the flg22 time course. Organized as in **Figure 9C**, for each individual DEG cluster from **Figure 8A**.
