# Supplementary material for: PAMP-triggered genetic reprogramming involves widespread alternative transcription initiation and an immediate transcription factor wave
Source: Plant Cell. 2022 Apr 11;34(7):2615–37. doi: 10.1093/plcell/koac108 (PMC9252474; doi:10.1093/plcell/koac108)
Supplement: koac108_Supplementary_Data [file tpc22.00124.PeerReviewReport.pdf › koac108-suppl_data/tpc22.00124.PeerReviewReport.pdf]

PAMP-triggered genetic reprogramming involves widespread alternative transcription initiation and an immediate transcription factor wave

Axel Thieffry, Diego López-Márquez, Jette Bornholdt, Mojgan Gholami Malekroudi, Simon Bressendorff, Andrea Barghetti, Albin Sandelin, and Peter Brodersen

Corresponding authors: Albin Sandelin (albin@binf.ku.dk) and Peter Brodersen (pbrodersen@bio.ku.dk)

Review timeline:

**TPC2021-LSB-00136**	Submission received:	Feb. 13, 2021
	1st Decision:	Feb. 18, 2021 *manuscript declined*
**TPC2021-LSB-00340**	Submission received:	April 15, 2021
	1st Decision:	June 5, 2021 *manuscript declined*
**TPC2022-RA-00124**	Submission received:	Feb. 3, 2022
	1st Decision:	Mar. 17, 2022 *accept with minor revisions*
**TPC2020-LSB-00124R1**	1st Revision received:	Mar. 22, 2022
	2nd Decision:	Mar. 24, 2022 *acceptance pending, sent to science editor*
	Final acceptance:	April 7, 2022

REPORT: (The report shows the major requests for revision and author responses. Minor comments for revision and miscellaneous correspondence are not included. The original format may not be reflected in this compilation, but the reviewer comments and author responses are not edited, except to correct minor typographical or spelling errors that could be a source of ambiguity.)

TPC2021-LSB-00136 1st Editorial decision – *declined* Feb. 28, 2021

Thank you for submitting your manuscript entitled "PAMP-triggered Genetic Reprogramming Involves Widespread Alternative Transcription Initiation and an Immediate Transcription Factor Wave" to The Plant Cell. Your submission has been evaluated by three members of the editorial board, and we concur that this is a very interesting dataset that significantly advances our understanding of PRR-Triggered Immunity (PTI). The finding of rapid changes in transcription start sites (TSSs) upon flg22 elicitation is particularly interesting. However, we expect that reviewers would request validation of the CAGE data by an independent assay to confirm the switch in TSSs. This would only need to be done for a small set of the most interesting genes, so hopefully would not require a significant amount of additional time. Related to this, it would strengthen the overall impact of this work if you could also show functional significance for at least one TSS-switched gene (e.g. MEKK1 could be analyzed by western blot and qRT-PCR to show a large increase in protein relative to RNA upon flg22 elicitation).

We feel it best to not send your paper out for review until you have had sufficient time to complete this work. However, we strongly encourage you to resubmit a revised version of your manuscript to The Plant Cell. Upon resubmission, we would immediately send it out for review.

TPC2021-RA-00340 1st Editorial decision – *declined* June 5, 2021

Thank you for choosing to send your manuscript entitled "PAMP-triggered Genetic Reprogramming Involves Widespread Alternative Transcription Initiation and an Immediate Transcription Factor Wave" for consideration at The Plant Cell. Your submission has been evaluated by members of the editorial board as well as expert reviewers in your field, and we regret to inform you that we are not able to recommend publication of this manuscript in its current form. However, we also agreed that if you could address the major points raised by the reviewers by additional analyses, we would welcome a resubmission. This may be treated as a new submission, but we would attempt to use at least some of the same reviewers. Nevertheless, reviewers will be asked to assess as a new manuscript (i.e. are the claims fully supported by the data and do the results presented move the field forward?), and not only whether previous reviewer comments have been addressed.

I ([Reviewing Editor]) have copied the verbatim reviews below. As you will read, Reviewers 1 and 2 were quite positive about the significance of this work, but both stated that the manuscript would be greatly strengthened by additional validation of two or more examples of interesting TSSs (e.g. by showing impact on subcellular targeting in a transient expression assay of the alternative isoforms). Reviewer 1 also pointed out that significant value would be added to this work by providing a more thorough comparison between the CAGE dataset and the standard RNA-seq dataset. I concur with these recommendations.

Reviewer 3 was decidedly more critical of the data generated using CAGE methods, and is especially concerned by the possibility that many of the TSS's identified by CAGE may not represent full length transcripts. It is not clear to me whether your inclusion of 5' RACE data can at least partially address this concern. If so, please comment on that (e.g. does the length of 5' RACE products establish a minimum length for these TSSs?), and/or provide additional data that would confirm that these TSS generate protein-coding transcripts. Also, I think the inclusion of additional functional validation on a couple more TSS's for interesting genes, as discussed in the previous paragraph, would help address this concern.

It will be important to convince the editors and reviewers that the major claims made are fully justified by the data presented. This includes careful consideration and explanation of the various controls used in experiments, the extent and manner of replication, and the statistical analyses used. Sampling methods and the nature of "biological replicates" should be described precisely (i.e. different plants, parts of plants, pooled tissue, independent pools of tissue, sampled at different times, etc.), along with a clear description of and rationale for any statistical analyses conducted. The reader should know exactly what was sampled; what forms the basis of the calculation of any means and other statistical variables and parameters reported. This is also necessary to ensure that proper statistical analysis was conducted.

--- Reviewer comments:

[Reviewer comments shown below along with author responses]

TPC2022-RA-00124 Submission received Feb. 3, 2022

Reviewer comments on previously declined manuscript and **author responses:**

We thank all reviewers for their input to our work. Overall, we find the points raised by reviewers 1 and 2 to be fair and constructive. We also believe that the concern of reviewer 3 on whether intragenic TSSs give rise to mRNAs encoding the polypeptides we discuss is fair. Thus, our revision efforts have focused on addressing the following two main points:

(1) Can we provide evidence that transcripts initiating from downstream TSSs produce stable, polyadenylated mRNAs that are translated?

(2) Can we provide evidence for functionality of at least some proteins encoded by such shorter isoforms, or of switches from short to long isoforms?

Regarding point (1): We now provide evidence that nearly all intragenic TSSs that we discuss in detail give rise to polyadenylated mRNAs with poly(A)-sites identical to those transcripts that arise from canonical upstream TSSs. This is based on two different lines of evidence that independently show the same results: i) original RIKEN Arabidopsis full-length cDNA data (Seki et al., 2002) and newly defined Arabidopsis full-length transcript assemblies combining Nanopore direct full-length RNA sequencing with several other data types (Ivanov et al., 2021). Thus, the main concern of reviewer 3 on whether the intragenic TSSs detected by CAGE that we discuss in detail represent noise transcripts is effectively countered. We do not, however, claim that *all* cases of alternative transcription initiation detected by CAGE necessarily have to be functionally important. Indeed, in the revised version we include additional analyses to show that the general trend across all multi-TSS genes is that the diversity of TSS usage decreases with gene expression level, an observation consistent with the view that many alternative TSSs could represent transcriptional noise, or at least RNAs with limited biological relevance.

Regarding point (2):

We have added much work in the present version to address this:

(i) We show that the long and short mRNA isoforms produced from the *PUS5* gene - one of our key examples - have very different properties: the long form encodes a protein that localizes to mitochondria and chloroplasts, while the short form encodes a nucleo-cytoplasmic protein. Therefore, the choice of which TSS is used (which in turn is treatment and time-specific in our experiment) confers a major functional difference (new Figure 5).

(ii) We show three cases in which the shift from long to short form results in marked translational activation by skipping of upstream open reading frames in the 5'-leader (new Figure 6). The shown cases are likely to be functionally relevant as they include an Hsp70 chaperone and its nucleotide exchange factor BAG6, and the protein kinase MEKK1, known to be important for signal transduction in PAMP-triggered immunity.

(iii) We show that the shift from the short to long form of the transcription factor HSFA7A is functionally relevant, because constitutive expression of the long form (otherwise only detected after flg22 treatment) is sufficient to cause growth restriction (new Figure 3).

Below, we provide a point-by-point explanation of how we have dealt with the criticisms of the reviewers.

Reviewer #1:

Reviewer 1, point 1: However, it is disappointing that the authors did not much compare CAGE and RNA-seq. This is doable and interesting for many readers. As many researchers perform RNA-seq but not CAGE, it is a great opportunity to compare two datasets generated in the same experimental setup and same time points. For example, the question that differentially regulated CAGE tag clusters are differentially regulated in RNA-seq and vice versa by visualizing together in a heatmap can be answered. Clustering of CAGE TCs and genes together and/or visualizing gene expression patterns along with existing CAGE TCs clusters would be informative. In addition, visualizing CAGE and RNAseq reads on genes using genome browser would give readers intuitive understanding of how CAGE and RNAseq reads are different or similar.

We agree that it is instructive to compare the CAGE and RNA-seq data obtained in this study. We now include an additional supplemental figure to show the RNA-seq signal at the genes with TSS switches that we discuss in detail (Supplemental Figure 5). This clearly illustrates how isoform switching at the TSS level is missed by RNA-seq, and we now include a small paragraph to comment on this fact. We also make use of the RNA-seq data to validate the overall temporal gene expression dynamics detected by CAGE, as in our originally submitted version (Supplemental Figure 7).

Reviewer 1, point 2: Another shortcoming is the lack of functional analysis of interesting alternative transcriptional initiation cases that the authors identified here. I would imagine that expressing different length of genes transiently in *Nicotiana benthamiana* followed by testing flg22 response (ROS, MAPK, bacterial resistance) would strengthen this manuscript. In addition, the authors also can easily express different length of genes fused to a fluorescent protein to investigate alternative protein localization.

As described above, we have taken this point of criticism very seriously, and now include functional analysis of several of long and short isoforms discussed in detail: We demonstrate isoform-dependent alternative PUS5 localization (organellar or nucleo-cytoplasmic, Figure 5), we demonstrate the importance of uORF skipping for BAG6, Hsp70, and MEKK1 (Figure 6), and we demonstrate that constitutive expression of the induced long isoform of HSFA7A is sufficient to cause growth restriction (Figure 3).

Reviewer 1, additional comments

I feel that introduction does not reflect the major findings of this manuscript. The authors spent eRNA and RNA decay but the authors did not find significance of those here. This confused me when reading this manuscript. The authors may shorten it. This is a suggestion but not request.

Our goal was to focus the introduction on the main points. In the end, the work includes CAGE profiling of nuclear exosome mutants, originally designed to search for PTI-related enhancers as explained. Since stress-responsive transcription has been shown in other organisms to involve nuclear exosomal decay it is also relevant to probe this pathway in stimulus-dependent transcriptional reprogramming in plants for the first time, as explained in the introduction. We have chosen not to change this dramatically, primarily because we have not encountered this criticism from other reviewers or colleagues who have read the manuscript.

I would imagine that promoter they defined and gene body are sometimes overlapped because TSS is very close to gene body. The authors should clarify this.

The use of these terms with respect to location of TC clusters in the present paper is defined in Figure 2B, and does not, in our view, require further clarification.

(Introduction) As far as I know, LPS receptor has not been characterized in plants.

An LPS receptor has been identified and is described in the following reference: A lectin S-domain receptor kinase mediates lipopolysaccharide sensing in *Arabidopsis* thaliana. Ranf S, Gisch N, Schäffer M, Illig T, Westphal L, Knirel YA, Sánchez-Carballo PM, Zähringer U, Hückelhoven R, Lee J, Scheel D. Nat Immunol. 2015 Apr;16(4):426-33. doi: 10.1038/ni.3124. Epub 2015 Mar 2.

Upon re-checking our manuscript, we have seen that the reference had been removed from a previous version by mistake. We have now re-inserted the reference to support the claim that an LPS receptor is one of the relatively few PAMP receptors that have been identified in plants. To be sure, we are aware that the examples mentioned in the introduction do not constitute a complete list.

(Introduction) I think that the authors should define what is "enhancers" as this term is not necessarily clear and different fields use it differently.

We have now briefly defined enhancers as distal regulatory regions that enhancer transcription initiation.

Page 2 Line 78-80. The logic is unclear to me.

Lines 78-80 read: "Specifically, the time gap between MAP kinase activation and documented transcriptional responses suggests that earlier changes in gene expression may be part of PTI activation."

In the section immediately preceding this statement, we have explained that MAP kinases phosphorylate, among other, transcription factors involved in PTI activation, that MAP kinases are activated within minutes (typically 2-5 minutes) of PAMP perception, and that changes in gene expression are typically measured 30 minutes after PAMP perception. Together, those facts motivate the need to profile gene expression changes much earlier than 30 minutes after PAMP perception, as explained in lines 78-80, and as done to great effect in the work we describe. We have now tried to make this even clearer by changing the sentence to:

"Specifically, the time gap between MAP kinase activation and documented transcriptional responses suggests that changes in gene expression occurring earlier than 30 minutes may be part of PTI activation."

Page 7 Line 202-204. What does "contributed 10% to their host gene expression" mean?

This simply means that the TC in question contributes at least 10% to the total expression of the gene (all CAGE tags mapping to the gene) to which it is assigned.

(Method) 1% MS medium is different from one that is used by many researchers. Is there any reason for it?

No - indeed, this is an error, and we thank the reviewer for spotting it. We used 1x MS medium, and the error has now been corrected in the two instances where it occurred.

<u>Reviewer #2:</u>

Reviewer 2, point 1 (1) I believe this work would be substantially strengthened if the functional relevance of PAMP-induced alternative transcription initiation change could be validated. For that purpose, in Figure 3F (At1g56345) and 3H (At1g67520), I suggest the subcellular localization of the longer and shorter protein isoforms should be examined in vivo, for example in transient expression system using particle bombardment.

We agree, and now provide evidence for the functional relevance of several cases of PAMP-induced TSS switching, including at the *PUS5* gene (AT1G56345) as suggested specifically by the reviewer. Other pieces of evidence are also included as described in the response to <u>Reviewer 1, point 2.</u>

Reviewer 2, point 2 2) In Figure 3H, the authors discussed the results as follows: "This pattern of re-focusing low-level noisy transcription initiation to a single, highly expressed TSS upon induction is probably an example of the recent proposition that alternative transcription initiation is often non-adaptive, and rather a consequence of imperfect TSS selection by the transcription initiation machinery" (line 376-380). However, I find myself in strong disagreement with this discussion because the possibility cannot be excluded that the shorter cytoplasmic protein isoform may have some unexpected adaptive function in the absence of PAMP.

We are also quite excited about this possibility, but decided to tone it down with the more "pessimistic" view that the lowly expressed TSSs detected could represent transcriptional noise. While we do think that the noise perspective is important to discuss, we agree that it is not well done by introduction of a specific example for which we actually do not know whether the alternative transcription initiation sites represent noise or production of functionally important forms. We have therefore removed the example of the lectin receptor-like kinase, and discuss alternative transcription initiation sites and noisy transcription more generally in relation to two new panels shown in Figure 2 (Figure 2F,G). These analyses show that diversity of TSS usage decreases with gene expression level.

<u>Reviewer #3:</u>

Reviewer 3, point 1: "These findings are very important, however I think CAGE data only used in this analysis is not enough to support their point. First, CAGE tags only indicate transcriptional start sites, not show and verify the whole transcripts. Sometimes it is associated with transcriptional noise and truncated transcripts that can not be elongated normally. So these incomplete transcripts are nonfunctional."

This is a fair point and we now show extensive evidence from RIKEN Full Length cDNAs, nanopore full-length RNA sequencing and Ribo-seq data that the large majority of the examples of alternative TSSs that we discuss define mRNAs with coding potential, often even with direct evidence that they can be translated. A more technical point here is that a large portion of prematurely terminated transcripts are short – less than 100 bp in both plants and animals (papers from Adelman and Marquardt groups) - and CAGE cannot detect these because of RNA size selection in the protocol. Moreover, truncated transcripts that are picked up by CAGE are typically exosome

substrates (see e.g. Wu et at, NAR 2020, PMID 3271063), and we see no evidence of their enhanced accumulation in mutants defective in nuclear exosome activity. This latter point has been incorporated into a new Supplemental Figure 2, and comments on its relevance have been introduced into the main text.

Reviewer 3, point 2: "Moreover, they do not show the proportion of one transcript with alternative TSS and normal TSS."

We have to disagree on this point. CAGE is, as a matter of fact, the most sensitive technique to provide quantitative information on TSS usage, and it specifically reports on the proportions of transcripts with TSS relative to another. The literature is rich in examples where CAGE has been used exactly for this purpose. In the present paper, we actually use its ability to detect proportions of transcripts with one TSS relative to another: we specifically state that we only discuss those TSSs that contribute at least 10% to the total transcript output from a gene. In the revised version, we have also added further analysis to explain to the reader why such a conservative expression threshold is important. We show that alternative TSS usage becomes less prevalent as expression level increases, suggesting that many cases of alternative TSSs could be regarded as transcriptional noise, and that introduction of expression thresholds, as done here, is necessary to identify the functionally important cases.

Reviewer 3, point 3: "I think that in the most time, transcripts with alternative TSSs have a very low expression level, of which are probably associated with transcriptional noise. The author must address it."

This is a sweeping criticism that implies that all alternative TSSs – including the ones we highlight as potentially important - are noise and that we did not consider expression levels.

It is certainly the case that alternative TSSs (or, indeed, even primary TSSs) can produce transcriptional noise, or just transcripts with low copy numbers, as described above. However, we have employed rigorous procedures, including conservative expression cutoffs and statistically significant differential expression which in turn also requires low variance, to identify those cases that are likely to be functional and we discuss several of those in detail in the paper. Others are listed in supplementary tables, easily searchable for interested readers. Therefore, the cases of interest that we discuss in detail cannot be considered to be "very low expression level".

Thus, the overall criticism here is not fair: while we do show that around 15% of all PAMP response genes display alternative transcription initiation, we are not claiming that all of these alternative TSSs produce functional products, but for those alternative PAMP-induced TSSs that we highlight as probably functional, the referee claim is simply not correct as we have already addressed it with rigorous criteria.

Reviewer 3, point 4: "Second, around the TSSs of genes, there are lots of miRNAs 30nt-200nt that are transcribed. Therefore, it is difficult to distinguish the CAGE signals that belong to alternative TSSs of a gene or TSS of miRNA."

We do not wish to be disrespectful of our colleague, but this is simply incorrect. First of all, microRNAs are not, as a matter of fact, transcribed from around TSSs of protein-coding genes. In Arabidopsis, most microRNAs are transcribed from intergenic regions, while some reside in introns of host genes, and very few are excised from exons of protein-coding transcripts. Therefore, the criticism is simply not valid.

The referee may possibly be referring to short RNAs produced by early RNA polymerase II termination events, as he/she did above. Such RNAs are typically extremely short-lived and require exosome mutants to be detected, as seen with the recent characterization of Short Promoter Proximal RNAs in Arabidopsis. As also mentioned above, a large proportion of such RNAs are also too short to be captured by CAGE.

Reviewer 3, point 5: "Based on these considerations, I think that CAGE is not good way to do this kind of study, unless RNA-seq data and full-length cDNA validation can be added into this work together."

CAGE is recognized by the field as the state-of-the art technique for finding TSSs. That said, we did already include RNA-seq data in the study. It is widely known that RNA-seq cannot accurately capture 5'- and 3'-ends of transcripts because of how the technique works: RNA fragmentation in combination with size selection will invariably result in lower read density at RNA ends. We observe this in the current study (however, the RNA-seq expression patterns nicely validates those of the CAGE data, as expected), and it is also in line with previous observations – for instance, the RNA-seq-based TSS annotation used in Araport11 is extremely inaccurate (Schon et al, Genome Research 2018, Thieffry et al, The Plant Cell 2020). Therefore, while we already include RNA-seq data in the manuscript, it is not the proper technique for TSS validation.

We do agree that validation from full length cDNA would strengthen the manuscript and as we described above, we now have evidence that most of the alternative TSSs of interest that we discuss do produce full-length transcripts, using full-length cDNA, Ribo-seq, and full length RNA from nanopore data.

Reviewer 3, point 6: "Additionally, this study has not yet set up control well. As far as I know, a great number of genes in Arabidopsis show alternative TSSs. Maybe, it is general or common phenomenon, but not specific to PAMP responses."

Which control does the reviewer have in mind? It would be technically impossible to rule out that a certain gene or TSS is affected *only* by PAMP and *no other* treatment, since the number of possible stimuli or treatments, and therefore experiments, is infinite. Moreover, we *do not* claim that alternative transcription initiation *only* happens upon PAMP perception – it is well known that alternative TSSs are important in other processes, for plants shown in responses to light, as we write in the introduction.

Here, PAMP-triggered immunity is used as an important, and easily controllable case of stimulus-dependent gene regulation, in this instance with the specific aim to study the extent and type of alternative transcription initiation and the speed of induced expression changes. We think that our study is very well designed to meet these aims, and that it contributes important new insights.

TPC2022-RA-00124 1st Editorial decision – *accept with minor revisions* Mar. 17, 2022

My deepest apologies for the long time in review, which was caused by a very tardy reviewer. We have finally received all reviews for your manuscript "PAMP-triggered Genetic Reprogramming Involves Widespread Alternative Transcription Initiation and an Immediate Transcription Factor Wave." On the basis of the advice received, the board of reviewing editors would like to accept your manuscript for publication in The Plant Cell. This acceptance is contingent on minor revisions to the text as recommended by Reviewer 1. comments (include only the customized paragraphs from decision letter).

-- Reviewer comments:

[Provided below along with author responses]

TPC2020-LSB-00124R1 1st Revision received Mar. 22, 2022

Reviewer comments on previous submission and **author responses**:

Reviewer #1

"Abstract: 'We show that around 15% of all PAMP response genes display alternative transcription initiation.' I don't think that the authors showed this data."

This relates to data shown in Figure panel 2C.

We thank the reviewer for spotting this mix--up of genes and CAGE tag clusters (TCs). We indeed show that, of all CAGE TCs induced during the PAMP response, close to 15% were alternative CAGE TCs. This averaged ratio originates from Figure 2C, where we display the different time point comparisons, e.g. 10 vs 0 min: 14.4%, 30 vs 0 min: 16.0%, and 30 vs 10 min: 15.3%. The abstract has been corrected accordingly.

"Regarding LPS receptor in plants. Ranf et al Nat Immunol 2015 proposed that LORE is the LPS receptor. However, later in Kutschera et al Science 2019, they found that LORE senses mc--3--OH--FA. While mc--3--OH--FA exists in commercial LPS and copurified with LPS, mc--3--OH--FA depleted LPS does not induce pathogen resistance. Thus, it appears that LORE senses mc--3--OH--FA but not LPS (Kutschera et al Science 2019)."

We thank the reviewer for drawing our attention to the 2019 paper that we had missed. We have now simply omitted the mention of LPS as an example of a PAMP for which a cognate receptor has been identified to leave the reader with three other such examples (flagellin, elongation factor Tu, chitin).

"I have still difficulty to understand "...a clear majority (90%) of detected genes had only one CAGE TC that contributed at least 10% to their host gene expression (this constraint was used for all intragenic TCs analyzed below, see Methods) (Figure 2A, Supplemental Dataset 1)." from the figure and dataset. 90% of genes contribute to 10% of gene expression?"

We have further clarified the text, so that it now reads as follows.

"Similar to our previous analysis on untreated seedlings (Thieffry et al., 2020), we found that most genes had only one CAGE TC: when we only considered CAGE TCs that contributed at least 10% to the expression of their cognate gene, 91% of all detected genes had only one CAGE TC (Figure 2A, Supplemental Dataset 1). This threshold of at least 10% contribution for a particular CAGE TC to be considered was used for all intragenic TCs analyzed below (see Methods)."

"Line 254 (ranks 2--6 shown in Figure 2F). ranks2--4?"

We thank the reviewer for spotting the error. We have corrected the text accordingly "(ranks 2--4 shown in Figure 2F)."

“Fig1C. The authors should describe the genotype used in the analysis in the figure legend.“

The genotype (wild type) has been added to the legend of Figure 1C.

“Fig3D. It would have been much better if the authors also analyzed the short isoform. I would expect them to do so in the future.”

We do not disagree with the reviewer that analyzing the flg22 response of plants constitutively expressing the short isoform of HSFA7A is an interesting experiment. We found that such plants trigger silencing from the 2nd to the 3rd transgenic generation at unusually high frequency, and a longer period of selection of appropriate transgenic lines, perhaps involving specific mutants in the RNA silencing machinery, is, therefore, required to conduct these experiments. This work is indeed on our wish list for the future, but will require additional financial support to be done.

“Fig5C and Line 419. The majority of the short form also accumulates in chloroplasts as the long form does. In addition, the short form may also accumulate in the nucleus.”

The reviewer is mistaken in this case. We specifically include images of leaf discs not expressing GFP to show to the reader that upon expression of the short PUS5--GFP isoform, the signal appearing in the green channel in chloroplasts is simply chloroplast autofluorescence. In contrast, nucleocytoplasmic signal in the green channel is specific to leaf discs expressing short PUS5--GFP. The red channel, otherwise irrelevant when studying GFP fluorescence, is also included precisely to make it easy for the reader to identify the green autofluorescence signal as originating from chloroplasts. All of this information is described in the legend. Thus, no modification of the description of the result or its interpretation is required here. Nonetheless, since even the reviewer did not get this part right, we think that it may also not be clear to many other readers. We have, therefore, made a simplified version of Figure 5C with only three images showing red and green channels overlaid on the DIC image: a negative control (red chloroplasts are evident), long PUS5--GFP(green chloroplasts are evident), short PUS5--GFP (green nucleus/cytoplasm and red chloroplasts are evident). Images of signal from the individual channels is shown in a new supporting Supplemental Figure 4 that allows the interested reader to see all details of why signal in the green channel is interpreted the way we do.

“Figure S5. The authors did not detect RNA--seq reads around some CAGE reads. For instance, there is no RNA--seq reads mapping to 5' HSFA7A mRNA at 30 min flg22 treatment. Does this mean that the long form of the transcript does not naturally exist at this time point even though the authors detected CAGE signals? I think that this should be discussed as they did around Line 545.“

We have expanded our discussion of mRNA--seq/CAGE discrepancy with the following note, inserted at line 507 in the original manuscript.

[The first word, “This”, refers to the requirement for poly(A) tails for detection in mRNA-- seq, but not in CAGE and 5’--RACE.]

“This may also explain specific discrepancies between CAGE and mRNA--seq in the examples studied here. For instance, although flg22--induction of the long isoform of HSFA7A is clearly detected by both CAGE and 5’--RACE at 30 mins after flg22 addition, mRNA--seq failed to reveal signal in regions specific to the long isoform in the 60--minute time course (Figure 3B, C;; Supplemental Figure 5).”

<u>OTHER CHANGES REQUIRED BY PLANT CELL STYLE</u>

Supplemental Figures

The current Supplemental Figure 6 is a small figure in its own right. It shows the mainly negative outcome of our searches for flg22--induced enhancer--like RNAs. We have, therefore, moved it to a main figure, so that it becomes main Figure 7. The manuscript now has a total of 9 main figures.

All other Supplemental Figures provide support and/or extensions to the main figures (e.g. additional controls, validations, full uncropped blots, documentation of transgenic line selection). In each case, we have stated clearly in the legends of the main figures where additional support can be found, and, vice versa, which main figure is supported by the supplementary figure in question.

Biological replicates

We added the following paragraph to the Methods section to make it clear to the reader what is meant by biological replicate in this study.

“Definition of Biological Replicates

Biological replicates were produced in the following way: Seeds from the same seed batch were germinated on different 1 x MS agar plates as described above, but grown in parallel and with plates next to one another in the same growth cabinet. Similarly, flg22--inductions were carried out separately for each replicate, with each replicate in a distinct well, but were done at the same time under the same growth conditions.”

Description of and rationale for statistical methods employed

We have not introduced additional changes in this regard, as we think that the current description in Methods is adequate.

TPC2020-LSB-00124R1 2nd Editorial decision – *acceptance pending* Mar. 24, 2022

We are pleased to inform you that your paper entitled "PAMP-triggered Genetic Reprogramming Involves Widespread Alternative Transcription Initiation and an Immediate Transcription Factor Wave" has been accepted for publication in The Plant Cell, pending a final minor editorial review by journal staff.

Final acceptance from Science Editor April 7, 2022
